# Adhesion and virulence properties of native *Metarhizium* fungal strains from Burkina Faso for the control of malaria vectors

**DOI:** 10.1186/s13071-023-05831-z

**Published:** 2023-11-07

**Authors:** Issiaka Sare, Francesco Baldini, Mafalda Viana, Athanase Badolo, Florencia Djigma, Abdoulaye Diabate, Etienne Bilgo

**Affiliations:** 1 Institut de Recherche en Sciences de la Santé, Direction Régionale de l’Ouest, BP 545, Bobo-Dioulasso 01, Burkina Faso; 2 Institut National de Santé Publique / Centre Muraz, BP 390, Bobo-Dioulasso 01, Burkina Faso; 3 Laboratoire d’Entomologie Fondamentale et Appliquée (LEFA), Université Joseph Ki-Zerbo, BP 7021, Ouagadougou 03, Burkina Faso; 4 Laboratoire de Biologie Moléculaire et de Génétique (LABIOGENE), Ecole Doctorale Sciences et Technologie, Université Joseph Ki-Zerbo, BP 7021, Ouagadougou 01, Burkina Faso; 5 Centre de Recherche Biomoléculaire Piétro Annigoni (CERBA), BP 364, Ouagadougou 01, Burkina Faso; 6 School of Biodiversity One Health and Veterinary Medicine, University of Glasgow, Glasgow, G12 8QQ UK

**Keywords:** *Metarhizium*, Entomopathogenic fungi, Malaria, Vector control, Burkina Faso

## Abstract

**Background:**

Local strains of the entomopathogenic fungus *Metarhizium pingshaense* in Burkina Faso have demonstrated remarkable virulence against malaria vectors, positioning them as promising candidates for inclusion in the future arsenal of malaria control strategies. However, the underlying mechanisms responsible for this virulence remain unknown. To comprehend the fungal infection process, it is crucial to investigate the attachment mechanisms of fungal spores to the mosquito cuticle and explore the relationship between virulence and attachment kinetics. This study aims to assess the adhesion and virulence properties of native *Metarhizium* fungal strains from Burkina Faso for controlling malaria vectors.

**Methods:**

Fungal strains were isolated from 201 insects and 1399 rhizosphere samples, and four strains of *Metarhizium* fungi were selected. Fungal suspensions were used to infect 3-day-old female *Anopheles coluzzii* mosquitoes at three different concentrations (10^6^, 10^7^, 10^8^ conidia/ml). The survival of the mosquitoes was measured over 14 days, and fungal growth was quantified after 1 and 24 h to assess adhesion of the fungal strains onto the mosquito cuticle.

**Results:**

All four fungi strains increased mosquito mortality compared to control (Chi-square test, χ^2^ = 286.55, *df* = 4, *P* < 0.001). Adhesion of the fungal strains was observed on the mosquito cuticle after 24 h at high concentrations (1 × 10^8^ conidia/ml), with one strain, having the highest virulent, showing adhesion after just 1 h.

**Conclusion:**

The native strains of *Metarhizium* spp. fungi found in Burkina Faso have the potential to be effective biocontrol agents against malaria vectors, with some strains showing high levels of both virulence and adhesion to the mosquito cuticle.

**Graphical Abstract:**

**Figure:**
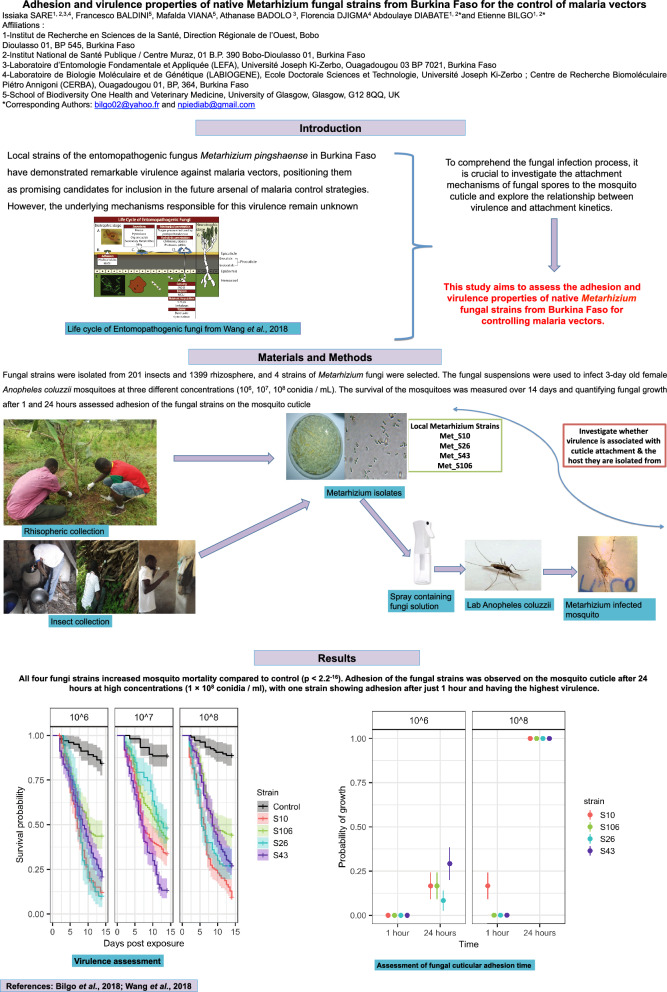

**Supplementary Information:**

The online version contains supplementary material available at 10.1186/s13071-023-05831-z.

## Background

Effective control of *Anopheles* vectors plays a critical role in malaria control programs [1]. However, despite the development of a large number of control tools against mosquito populations, the current vector control strategies implemented in most African countries have not been sufficient to block malaria transmission [2]. In Africa, most national malaria vector control programs still rely only on indoor residual spraying (IRS) and long-lasting insecticidal mosquito nets (LLINs). However, the effectiveness of LLINs and IRS is threatened by residual transmission as well as by increasing levels of insecticide and behavioral resistance in target vectors [3]. In order to provide an effective response to the elimination of malaria, WHO suggests using the full range of vector control measures as complementary tools, but given the level of residual transmission, it is crucial to continue developing new vector control strategies. One promising tool is the use entomopathogenic fungi [4, 5].

Unlike bacteria and viruses, entomopathogenic fungi have the ability to infect their host through tarsal contact. Although fungal entomopathogens are taxonomically very diverse, they all have the ability to produce infectious spores which attach to, germinate and enter the epicuticle of their host [6]. The first phase of infection is the attachment of the spores to the mosquito cuticle through hydrophobins, which constitute a layer of rods contributing to the initial hydrophobicity of the cell surface [7]. The growth of appressoria, which are swollen plugs from which tiny infection pegs enter the cuticle using a combination of mechanical force and cuticle-degrading enzymes, and germ tubes produce a sticky mucilage that helps localize the secreted enzymes, degrading the cuticle [8]. In infections of the fungus *Metarhizium* spp., the appressorium is elicited in response to hydrophobicity, insect cuticle surface structure and analysis of signals from the insect, such as nutrient levels and patterning of cuticle molecules; these signals allow *Metarhizium* spp. fungi to determine if they are on an appropriate host cuticle [7].

There are a wide range of entomopathogenic fungi, and many, especially local fungi strains [9]**,** have been proposed as a biological control agent. Compared to imported fungal strains, native fungal isolates may offer a better alternative for biological control of vectors as they may be better adapted to both kill local insects and survive local environmental conditions [4]. The fungal genus *Metarhizium* is found naturally in several host ranges, including plants and insects, and in several forms of biological associations, including symbiosis and parasitism [10]. In addition, members of the genus *Metarhizium* associate with plant roots where they influence the growth of plant hormones [11]. In Burkina Faso, the use of a local fungus capable of controlling mosquito populations has been investigated [4]. As such, the discovery of local fungi from the same environment would be a considerable asset because this type of fungi could tolerate well the local environment (temperature, humidity). The first key step to start developing this tool is therefore to isolate and assess the capacity of local fungi against malaria vectors.

The two strains of *Metarhizium pingshaense* fungus isolated from Burkina Faso were named S10 and S26 and have been shown to effectively kill mosquitoes [4]. However, the relationship between virulence and cuticle contact time has never been elucidated. The expression of virulence in the fungus is a cascade of mechanical (pressure) and chemical (production of enzymes) interactions [12]; in fact, insect cuticle recognition and infection are the first steps of fungal infection within mosquitoes. The kinetics of this infection may have an important impact on fungal virulence between strains, and understanding the physiological biochemical mechanism of fungal attachment and penetration of the fungi to the mosquito cuticle can help in the selection of the most virulent strains for vector control applications.

Additionally, the relationship between the origin of the fungus and its virulence on its host has not been sufficiently studied. It is therefore necessary to investigate the virulence of the fungus collected from the mosquito and compare it to that of fungi collected from the rhizosphere, in order to understand if the virulence of fungal strains could be increased by adaptation to a specific host. If so, selecting fungal strain directly from mosquitoes could offer new opportunities, as strains originally isolated from other sources (e.g. roots) could be optimized for vector control by in vivo culturing on mosquitoes. The primary goal of the present study was to isolate additional local native strains of* Metarhizium* that can rapidly kill mosquitoes and then investigate whether virulence is associated with cuticle attachment and the host from which they were isolated. These insights would inform the best practice to select effective entomopathogenic strains against malaria vectors.

## Methods

### Study site

**Fig. 1:**
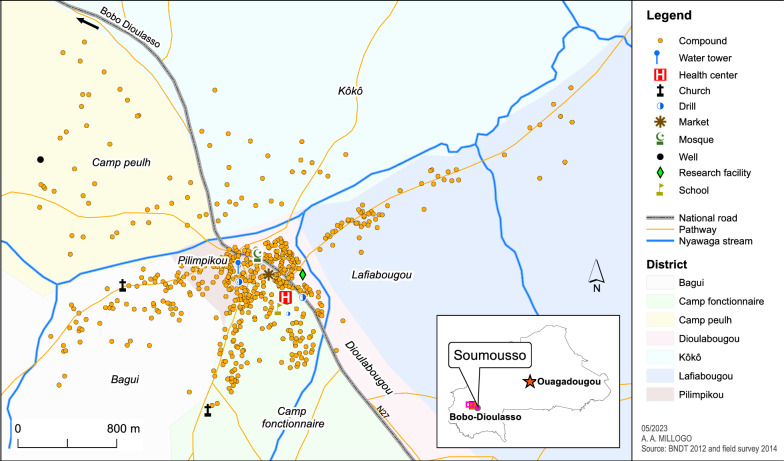
Map of Soumousso village. The collection took place in households, abandoned houses and fields at the entrance to and exit from the village

Collections of crawling insects and rhizospheres (region of the soil directly formed and influenced by the roots and associated micro-organisms that are part of the plant microbiota) were carried out from October to December 2018 in village of Soumousso village, western Burkina Faso (11° 04′N, 4° 03′W) (Fig. 1; Additional file 1: Table S1).

### Selective isolation of fungal strains

Crawling insects in inhabited houses, uninhabited houses and plant roots (specifically the plant parts contained in the first soil horizon [the rhizosphere]) were collected. The roots of plants, which present some nodes on their upper parts, were collected, and fungi were isolated using standard protocols as previously described [13]. Briefly, the collected insects and plants were crushed using a manual grinder in 50-ml tubes, with each tube containing 50 µl of Tween 80%. This fungal solution was then placed in Petri dishes containing a fungal selective medium (per liter: 42 g of potato-dextrose agar [PDA], 0.5 g of chloramphenicol and 0.6 g of cetyltrimethylammonium bromide [CTAB]) for growth. Despite using this selective media, during growth there was frequently a mixture of one or more strains of fungus on the same Petri dish. This necessitated that a purification step be carried out on Petri dishes in which a colony mixture was observed. Colonies were observed as a function of coloration during successive subculturing, until a single and uniform colony color was obtained on the Petri dish. Fungal isolates of *Metarhizium* were identified according to macro- and micro-morphological characters, such as conidiogenesis, spore color and mycelial texture of isolates on the PDA medium [14]. Met_S10 and Met_S26 were confirmed as *Metarhizium pingshaense* by amplification and Sanger sequencing of the intron-rich region of translation elongation factor 1-α [14]**.**

### Macroscopic characterization of* Metarhizium* spp.

Macroscopic observation of entomopathogenic fungi is a method widely used to characterize fungal strains [15]. Macroscopic characterization was carried out according to the technique described in [16]. Mycelial fragments were taken from the Petri dishes containing the antibiotics and the fungicides using a sterile spatula at the level of the growth front and placed on PDA culture medium in Petri dishes, then incubated in the dark at 27 °C ± 2 °C for approximately 21 days. Some clues that were considered indicative of the identity of strains of *Metarhizium* spp. were observed with the naked eye, including the presence or absence of droplets on the back of the Petri dish, production of diffusible pigment, color of the aerial mycelium and its variation over time, among others.

### Microscopic characterization of* Metarhizium* fungi

Observations were made on the basis of the classification according to the morphological criteria [16] for the identification of genus *Metarhizium* spp. This consisted of taking a fragment of the thallus of each isolate using a sensor, then depositing this fragment into a drop of sterile distilled water onto a slide and covering it with a coverslip. The slide with specimen was then observed under a optical microscope (magnification 40×) to identify organs, as indicated in [17], by careful observation of the morphology of spores, conidiophores and phialides.

### Molecular identification of* Metarhizium* fungi

Molecular identification of *Metarhizium* spp. was performed in two steps: DNA extraction and the amplification of the nuclear ribosomal internal transcribed spacer (ITS) region. First, the mycelium of each strain was harvested by scraping with a sterile spatula in a laminar flow hood. DNA was extracted using the 2% CTAB procedure [18]. PCR analyses of the ITS region were performed as described in [16] using primers ITS1 (5′-TCCGTAGGTGAACCTGCGG- 3′) and ITS4 (5′-TCCTCCGCTTATTGATATGC-3′). The reaction mix (total volume: 20 μl) contained 4 μl PCR Mix (5×; Solis Biodyne, Tartu, Estonia), 1 μl ITS1 primers (10 μM), 1 μl ITS4 primers (10 μM), 2 μl DNA and 12 μl PCR water. The cycling program consisted of an initial denaturation at 95 °C for 10 min, followed by 45 cycles of denaturation at 95 °C (10 s), hybridization at 58 °C (10 s) and elongation at 72 °C (40 s); with a final additional elongation at 72 °C (5 min). The negative control did not contain any fungal DNA. The reactions were loaded in a 1.5% agarose gel prepared with Invitrogen™ 10 mM Tris–HCl buffer (pH 8.0) (Invitrogen, Thermo Fisher Scientific, Waltham, MA, USA) and run in an electric field at 70 V for 45 min, following which the gel was observed using a digital imaging system under UV light. Fragment sizes were measured using an Invitrogen 100-bp molecular weight marker. The expected band size for our samples was 550 bp.

### Preparation of fungal solutions

The solutions containing the different strains were formulated as described previously [4]. Briefly, the fungi cultivated on PDA for a period of at least 2 weeks (to obtain mature spores) were harvested and then immersed in Tween 80% solution. The solution obtained was vortexed to obtain a homogeneous solution and then diluted 1:10 to calculate the number of spores by counting them under the microscope. To estimate the number of spores received by each mosquito after spraying, three mosquitoes from each batch were placed in 100 µl of distilled water and vortexed for about 15 s; the solution was diluted 1:10, and spores in the diluted solution were counted under the microscope. Each mosquito received approximately 15, 19 and 23 conidia, achieving concentrations of 1 × 10^6^, 1 × 10^7^ and 1 × 10^8^, respectively (Supplementary Figure 2).

### Mosquito rearing

*Anopheles coluzzii* mosquitoes were collected at Vallee du Kou (11°23'N, 4°24'W) and brought to the insectarium for rearing. Larvae, pupae and adults were reared and maintained under standard conditions of 27 ± 2 °C and 80 ± 10% relative humidity with a light/dark cycle of 12/12 h.

### Fungal virulence bioassay

Adult *An. coluzzii* mosquitoes aged 3 to 5 days were exposed to local fungal isolates of* M. pingshaense* strains S10, S26 [4], S43 and S106 following the protocol described in [16]. Briefly, mosquitoes were distributed in batches of 20 mosquitoes per cup, the cups with mosquitoes were exposed to − 20 °C for 15 s to anesthetize the mosquitoes, the mosquitoes were then transferred to a Petri dish lined with filter paper and, finally, the mosquitoes were sprayed with the fungal solutions using a small sprayer.

Four* Met. pingshaense* strains (S10, S26, S43, S106) and three concentrations (1 × 10^6^, 1 × 10^7^ and 1 × 10^8^ conidia/ml) were used, with Tween 80% being used as the solvent. Control mosquitoes were exposed to a solution of 80% Tween only. Twice-daily counts of dead mosquitoes were performed for a period of 2 weeks following exposure to the fungal solution. A mosquito was classified as dead if it was still, could not stand or showed no signs of life.

### Fungal growth assessment on cadavers after bioassays

The death of the mosquito due to the action of the fungus was confirmed 5 days post exposure if *Metarhizium* fungal growth was identified on the body of the dead mosquito. Specifically, dead mosquitoes were collected and washed with 2% bleach for 15 s and rinsed with sterile distilled water for 30 s. The dead mosquitoes were then placed on agar-based potato medium incubated at 27 °C for a period of 5 days, after which the fungal growth on the dead mosquitoes was observed; mosquitoes showing the presence of fungal growth were those considered to have died from the infection.

### Cuticle attachment assay

To assess cuticle attachment time, mosquitoes were exposed to the control or a fungal solution and then killed after 1 to 20 h. The freshly killed mosquitoes were then immediately washed with 2% bleach for 15 s and rinsed with sterile distilled water for 30 s, placed on agar-based potato medium and incubated at 27 °C; after 5 days of incubation the dead mosquitoes were analyzed for fungal growth.

### Fungal infection of mosquitoes through contact between dead and live mosquitoes

Live mosquitoes were exposed to mosquitoes that had died from the infection. Specifically, live mosquitoes were first immobilized by placing them in the freezer for 15 s, then bringing them into direct contact with 10 dead mosquitoes infected with fungi, in a Petri dish. Live mosquitoes were then released into cages (15 × 15 × 15 cm). Mosquito survival was measured for 2 weeks by counting individual deaths twice daily; these values were compared to the number of deaths of mosquitoes that were in contact with fungi isolated from the soil.

### Data analysis

All data were entered in Microsoft Windows Excel 2010 (Microsoft Corp., Redmond, WA, USA) and then analyzed using R version 4.1.2 (R Foundation for Statistical Computing, Vienna, Austria). Three models were developed to determine: (i) the impact of the different fungal strains and of the different fungal concentrations on mosquito survival; (ii) the survival of mosquitoes exposed to fungal strains isolated either from the soil or from mosquito carcasses; and (iii) the association between fungal growth and different strains at different time points (1 and 24 h post infection). For the first two survival models, a Cox proportional hazard model from the R package 'survival' was used, incorporating the random effect of 'replicate' by fitting a frailty function [19]. The fixed effects for the first model were strains and concentration and for the second, strains and origin (soil or mosquito). For both of these models, the best model was identified on the basis of analysis of variance (ANOVA), where a maximal statistical model including all fixed effects separately and as an interaction, as well as random effects was created, and then non-significant terms were sequentially removed to obtain a final model containing only the statistically significant predictors [20]. For the analysis of fungal growth, the generalized linear mixed model (GLMM) in the R statistical package 'glmer' was used [20]. Specifically, a binomial model with a binary response variable of growth (yes/no) was created with strain, time, concentration and an interaction between strain and time added as fixed effects and replicate as a random effect. Non-significant terms were sequentially removed following the backward elimination procedure as described above.

## Results

### Isolation of native* Metarhizium *strains

A total of 1600 samples of both arthropods (insects) and plant tissues associated with the rhizosphere were collected. Of these, 201 were identified as live or dead insects (mostly Coleoptera) and 1399 were plant tissues (roots and bark). All samples were processed to identify the range of possible fungi, including species of genera *Beauveria*, *Trichoderma*, *Fusarium* and *Aspergillus*. From these samples we isolated two new *Metarhizium* strains, which we named S43 and S106.

### Quantification of virulence against malaria mosquitoes

**Fig. 2:**
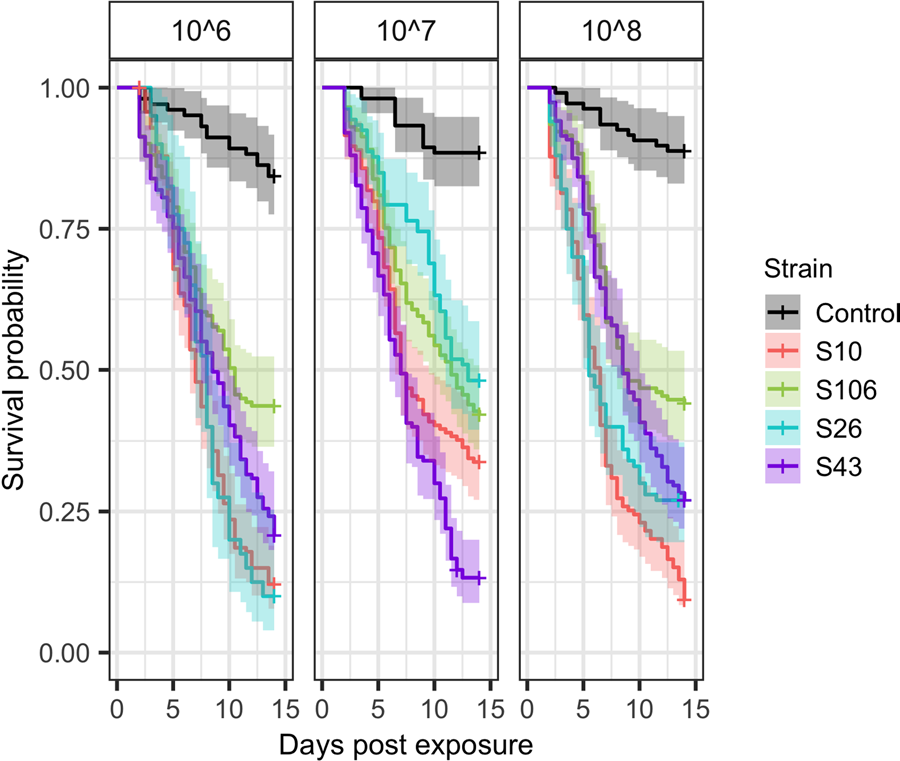
Virulence assessment. Survival curves of mosquitoes exposed to different* Metarhizium* strains and control at different concentrations over a 2-week period after exposure. Solid lines represent the predicted mean survival and the shaded areas show the 95% confidence interval

Female adult mosquitoes of *Anopheles coluzzii* were exposed to four native strains of *Metarhizium* sp. from Burkina Faso (S10, S26, S43 and S106) at three different concentrations (1 × 10^6^; 1 × 10^7^; and 1 × 10^8^ conidia/ml) and their virulence measured by monitoring mosquito survival for 2 weeks. All four fungal strains resulted in a higher mosquito mortality compared to the control, as depicted in Fig. 2 (Chi-square test, χ2 = 286.55, df= 4, P < 0.001). The survival of mosquitoes was significantly associated with an interaction between strain and concentration (Additional file 1: Table S2) (Chi-square test, *χ*^2^ = 54.424, *df* = 4, * P* < 0.001). All four strains were observed to cause significant mortality and, generally, increased concentrations were associated with increased mortality (Fig. 2).

### Measurement of cuticle adhesion time

**Fig. 3:**
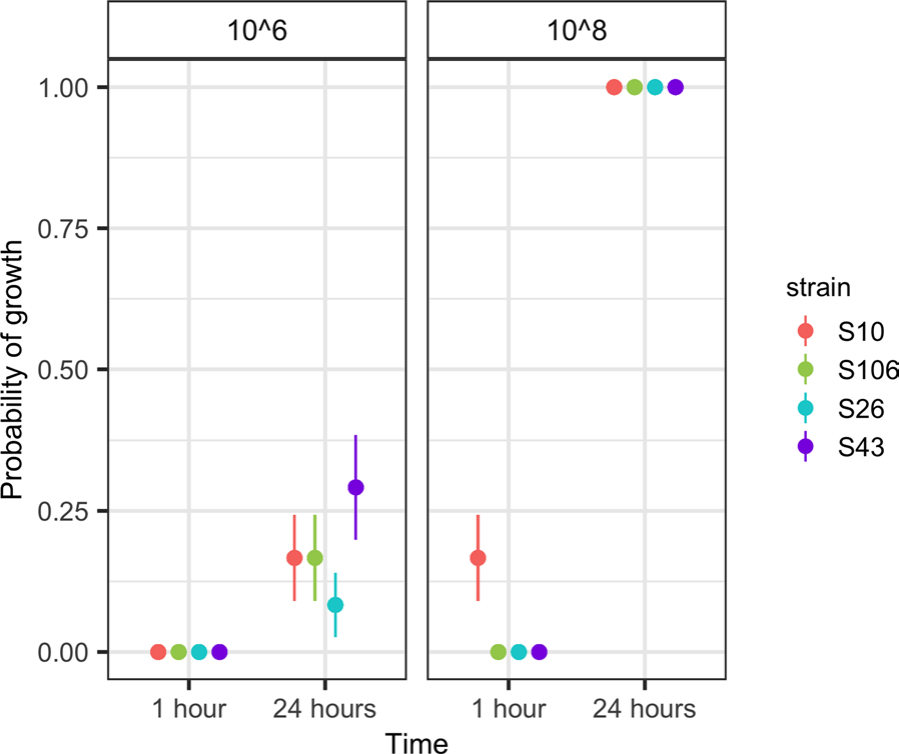
Assessment of fungal cuticular adhesion time. Probability of fungal growth at 1 and 24 h after exposure at two concentrations (10^6^ and 10^8^ conidia/ml) of the four * Metarhizium* strains. The filled circle represents the predicted mean probability of growth and the line shows the 95% confidence interval

The ability of strains to adhere to the mosquito cuticle was analyzed by measuring fungal growth on mosquitoes 1 and 24 h after exposure to the fungi under two different concentrations (1 × 10^6^; 1 × 10^8^ conidia/ml). The interaction between fungal strain and both time (Deviance, *D* = − 9.779, *P* = 0.021) and concentration (Deviance, *D* = − 178.93, *P* < 0.001) were significantly associated with fungal growth (Additional file 1: Table S3). At both concentrations all strains adhered to the mosquito cuticle after 24 h, although adherence was only partial at the lowest concentration (Fig. 3). When mosquitoes were exposed to the lower concentration (1 × 10^8^ conidia/ml), only the S10 strain adhered to the cuticle as quickly as 1 h after exposure (Fig. 3); this same strain was also the most virulent at this concentration (Fig. 2), possibly suggesting an association between adhesion and virulence. Another strain (S43) was relatively capable of adhering to the mosquito cuticle at the lowest concentration (Fig. 3).

### Virulence and fungal origin

**Fig. 4:**
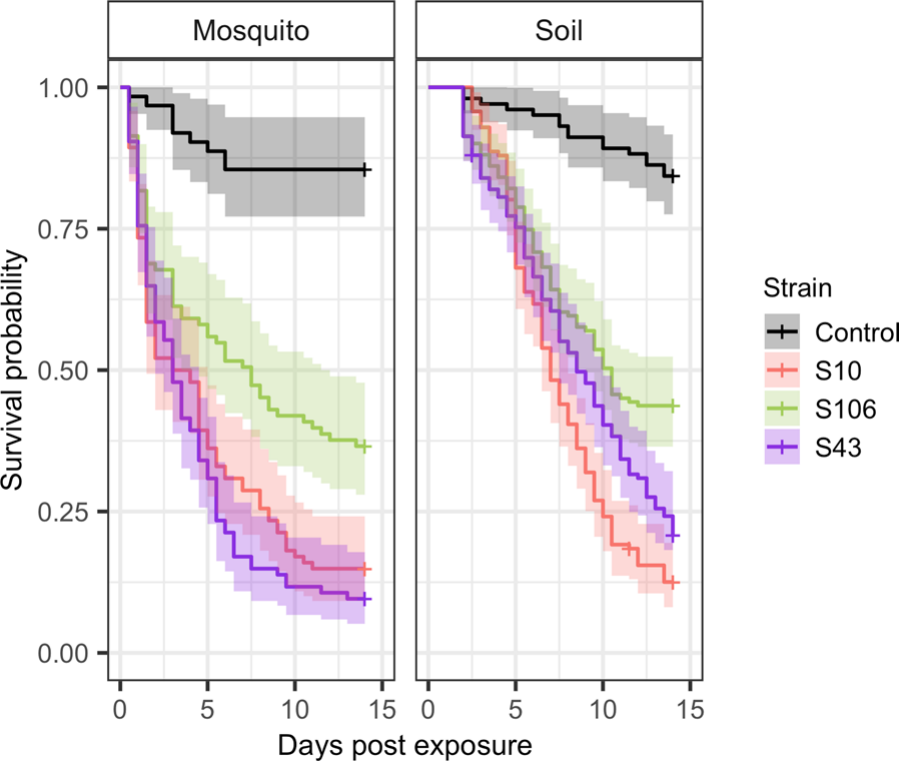
Mortality of malaria mosquitoes exposed to strains of the wild fungus *Metarhizium*. Survival curves of mosquitoes exposed to different strains of fungi from different origins (soil and host) and of a group of control mosquitoes up to 2 weeks after exposure. Solid lines represent predicted mean survival and the shaded areas show the 95% confidence intervals

The influence of the origin of the fungi on virulence was tested by exposing mosquitoes to fungi isolated either from the soil or from dead mosquitoes at the highest concentration of 1 × 10^8^ conidia/ml. A significant interaction between strain and origin was found (Chi-square test, *χ*^2^ = 12.078, *df* = 4, *P* = 0.021), confirming that all four strains caused significant mortality compared to the control (Additional file 1: Table S4). Additionally, exposure to fungi previously isolated from *Metarhizium-*infected mosquitoes significantly increased mortality compared to fungi isolated from the environment (Fig. 4).

## Discussion

The results of this study revealed the competence of different *Metarhizium* strains isolated from the field to kill mosquitoes collected in the wild and brought back to the laboratory. In addition to the strains previously identified [4] (S10 and S26), we also identified two additional strains (S106 and S43) capable of killing mosquitoes. These new strains decimated the mosquitoes after 3 days, with increasing mortality dependent on the concentrations of the fungal solution. The mosquitoes received approximately 15 and 23 spores when exposed to concentrations of 10^6^ and 10^8^ conidia/ml, respectively, suggesting that spore density is an important factor in the virulence of the fungus. As fungal strain is evaluated based on the number of viable spores within it, for the implementation of these fungi in the field, the final concentration must be defined and then the most virulent strain selected. All strains tested in this study (old and new) killed > 50% of the mosquito populations within 10 days post-infection. These fungal strains were able to kill more than half of the mosquitoes regardless of their origin; however, the post-infection mortality rate was higher for the strains isolated from the host.

Adhesion is one of four important phases in the infection process of agents that infect by contact [21]. This study has shown that the different strains of fungi did not achieve significant adhesion after 1 h of contact regardless of the fungal concentration. However, strain S10 at the concentration 10^8^ conidia/ml showed partial adhesion quickly after 1 h of contact, while the other strains showed some level of adhesion only after 24 h of infection. This result suggests that most of the isolated fungi need > 1 h to establish adhesion structures such as the appressoria [7] and, thereby, to express their virulence on the host population. Additionally, adhesion time might be associated with mechanical and enzymatic events, such as the penetrating pressure of the growing hyphae and the secretion of fungal proteases [7], as well as exposure of a specific adhesin-like protein (Mad1) following spore expansion [12]. As most entomopathogenic fungi of the genus *Metarhizium* use a combination of mechanical force, cuticle-degrading enzymes and toxins to facilitate penetration into the body of its host [22], future studies should investigate the mechanism used by the isolated fungal strains to penetrate the mosquito host. This result also suggests a possible association between virulence and cuticular adhesion, opening research lines for new studies, such as an investigation of whether faster cuticle adhesion is linked to an increased virulence.

We showed that the strains are more virulent when isolated immediately from an ecologically close host. Indeed, fungi taken directly from dead mosquitoes were found to be more virulent than those isolated from the soil. This could be explained by the establishment of specific infectious structures during the infection of mosquitoes. The infection of a range of insects by the fungus makes certain structures initially naïve to specialize in this specific type of host [23]. Alternatively, it could be that the amounts of spores that mosquitoes were exposed to between soil-isolated and dead infected mosquitoes were different, thus resulting in different virulence. Future studies should aim at quantifying the spores that mosquitoes acquire when in contact with dead mosquitoes.

Based on the results of this study, it appears that fungal virulence and cuticular adhesion of *Metarhizium* species are not isolated events, and that the faster the fungus adheres to the cuticle, the less time the mosquito has to develop means of avoidance. Cross infection between individuals in the wild has also be observed [24], suggesting that this type of infection by cuticular contact between dead and live mosquitoes could be exploited to disseminate the fungus in the wild.

## Conclusions

This study focused on the use of entomopathogenic fungi as agents for malaria vector control, with a particular emphasis on locally isolated strains that are better adapted to local climatic conditions. Findings from the current study suggest that application of *Metarhizium* at a moderate concentration can effectively infect mosquitoes and significantly increase mortality rates. The primary effect of the fungus in control programs is to reduce the probability of mosquito survival, which in turn reduces the likelihood of an infected mosquito reaching the infective state, ultimately decreasing the risk of malaria transmission. As kinetics and cuticular adhesion are key events related to the virulence of the fungus, future studies should investigate the genes involved in these phenomena. The native *Metarhizium* fungal strains found in Burkina Faso have demonstrated high virulence and adhesion to the mosquito cuticle, indicating their potential as effective biocontrol agents against malaria vectors.

### Supplementary Information

**Additional file 1: Table S1.** List of fungal isolates collected. **Figure S1.** Number of spores that individual mosquitoes received for three different concentrations. **Figure S2.** Macroscopic and microscopic features of native strains of* Metarhizium pingshaense* from Burkina Faso. **Table S2.** Statistics for the model on survival after fungal exposure. **Table S3.** Statistics for the model on association between fungal exposure time and strain. **Table S4.** Statistics for the model on survival after exposure to soil isolated fungi or fungal infected mosquitoes.

## Data Availability

The authors declare that the data supporting the findings of this study are available within the paper and its supplementary files.

## Abbreviations

Met_S10: *Metarhizium pingshaense* strain no. 10
Met_S26: *Metarhizium pingshaense* strain no. 26
Met_S43: *Metarhizium* sp. strain no. 43
Met_S106: *Metarhizium* sp. strain no. 106
