# Supplementary material for: Adhesion and virulence properties of native Metarhizium fungal strains from Burkina Faso for the control of malaria vectors
Source: Parasit Vectors. 2023 Nov 7;16:406. doi: 10.1186/s13071-023-05831-z (PMC10629044; doi:10.1186/s13071-023-05831-z)
Supplement: Supplementary file 1 — Additional file 1: Table S1. List of fungal isolates collected. Figure S1. Number of spores that individual mosquitoes received for three different concentrations. Figure S2. Macroscopic and microscopic features of native strains of Metarhizium pingshaense from Burkina Faso. Table S2. Statistics for the model on survival after fungal exposure. Table S3. Statistics for the model on association between fungal exposure time and strain. Table S4. Statistics for the model on survival after exposure to soil isolated fungi or fungal infected mosquitoes. [file 13071_2023_5831_MOESM1_ESM.docx]

**Supplementary Material**

Native *Metarhizium* strains: kinetics of adhesion to the cuticle and their virulence against mosquitoes

Issiaka SARE^1, 2,3,4^, Francesco BALDINI^5^, Mafalda VIANA^5^, Athanase BADOLO ^3^, Florencia DJIGMA^4^ Abdoulaye DIABATE^1, 2^*and Etienne BILGO^1, 2^*

Affiliations :

1-Institut de Recherche en Sciences de la Santé, Direction Régionale de l’Ouest, Bobo

Dioulasso 01, BP 545, Burkina Faso

2-Institut National de Santé Publique / Centre Muraz, 01 B.P. 390 Bobo-Dioulasso 01, Burkina Faso

3-Laboratoire d’Entomologie Fondamentale et Appliquée (LEFA), Université Joseph Ki-Zerbo, Ouagadougou 03 BP 7021, Burkina Faso

4-Laboratoire de Biologie Moléculaire et de Génétique (LABIOGENE), Ecole Doctorale Sciences et Technologie, Université Joseph Ki-Zerbo ; Centre de Recherche Biomoléculaire Piétro Annigoni (CERBA), Ouagadougou 01, BP, 364, Burkina Faso

5-School of Biodiversity One Health and Veterinary Medicine, University of Glasgow, Glasgow, G12 8QQ, UK

*Corresponding Authors: [bilgo02@yahoo.fr](mailto:bilgo02@yahoo.fr) and [npiediab@gmail.com](mailto:npiediab@gmail.com)

**Supplementary Table 1. List of fungal isolates collected**. ND = Not Determined.

| **N.of isolates** | **Host-name** | **Type collection** | **Site** | **Date of collection** | **Fungal species** |
| --- | --- | --- | --- | --- | --- |
| 5 | Cassia siamea | Rhizosphere | Soumousso | October-2018 | Trichoderma spp |
| 1 | Phragmites australis | Rhizosphere | Soumousso | October-2018 | Beauveria spp |
| 1 | Khaya senegalensis | Rhizosphere | Soumousso | October-2018 | Beauveria spp |
| 2 | Azadirachta indica | Rhizosphere | Soumousso | October-2018 | Beauveria spp |
| 2 | Sclerocarya birrea | Rhizosphere | Soumousso | October-2018 | Beauveria spp |
| 2 | Thevetia neriifolia | Rhizosphere | Soumousso | October-2018 | ND |
| 1 | Adansonia digitata | Rhizosphere | Soumousso | October-2018 | Beauveria spp |
| 3 | Bombax costatum | Rhizosphere | Soumousso | October-2018 | Beauveria spp |
| 1 | Zea mays | Rhizosphere | Soumousso | October-2018 | Aspergilus |
| 6 | Sorghum bicolor | Rhizosphere | Soumousso | October-2018 | Aspergilus |
| 6 | Sesamum indicum | Rhizosphere | Soumousso | October-2018 | ND |
| 2 | Nicotiana tabacum | Rhizosphere | Soumousso | October-2018 | Aspergilus |
| 2 | Tectona grandis | Rhizosphere | Soumousso | October-2018 | Beauveria spp |
| 2 | Vitellaria paradoxa | Rhizosphere | Soumousso | October-2018 | Trichoderma spp |
| 2 | mangifera indica | Rhizosphere | Soumousso | October-2018 | Trichoderma spp |
| 1 | Phaseolus vulgaris L | Rhizosphere | Soumousso | October-2018 | Trichoderma spp |
| 1 | Lannea microcarpa | Rhizosphere | Soumousso | October-2018 | Trichoderma spp |
| 5 | Balanites aegytiaca | Rhizosphere | Soumousso | October-2018 | Beauveria spp |
| 6 | Saba senegalensis | Rhizosphere | Soumousso | October-2018 | Trichoderma spp |
| 1 | Nauclea latifolia | Rhizosphere | Soumousso | October-2018 | Trichoderma spp |
| 1 | wild grass | Rhizosphere | Soumousso | October-2018 | Trichoderma spp |
| 4 | Lannea microcarpa | Rhizosphere | Soumousso | October-2018 | Trichoderma spp |
| 3 | Sesamum indicum | Rhizosphere | Soumousso | October-2018 | Aspergilus |
| 1 | Vigna subterranea | Rhizosphere | Soumousso | October-2018 | Metarhizium spp |
| 1 | Vitellaria paradoxa | Rhizosphere | Soumousso | October-2018 | Metarhizium spp |
| 3 | Solanum lycopersicum | Rhizosphere | Soumousso | October-2018 | Metarhizium spp |
| 1 | Thevetia neriifolia | Rhizosphere | Soumousso | October-2018 | Fusarium spp |
| 3 | Bombax costatum | Rhizosphere | Soumousso | October-2018 | Fusarium spp |
| 1 | Lagenaria siceraria | Rhizosphere | Soumousso | October-2018 | Fusarium spp |
| 1 | Jatropha curcas | Rhizosphere | Soumousso | October-2018 | Aspergilus |
| 4 | Hibiscus rosa-sinensis | Rhizosphere | Soumousso | October-2018 | Aspergilus |
| 1 | Balanites aegyptiaca | Rhizosphere | Soumousso | October-2018 | Aspergilus |
| 2 | Zea mays | Rhizosphere | Soumousso | October-2018 | Aspergilus |
| 1 | Nicotiana tabacum | Rhizosphere | Soumousso | October-2018 | Aspergilus |
| 6 | Capsicum frutescens | Rhizosphere | Soumousso | October-2018 | Trichoderma spp |
| 1 | Sorghum bicolor | Rhizosphere | Soumousso | October-2018 | Trichoderma spp |
| 3 | Dioscorea alata | Rhizosphere | Soumousso | October-2018 | Trichoderma spp |
| 1 | Gossypium barbadense | Rhizosphere | Soumousso | October-2018 | Trichoderma spp |
| 4 | Vitex donania | Rhizosphere | Soumousso | October-2018 | Trichoderma spp |
| 2 | Arachis hypogaea | Rhizosphere | Soumousso | October-2018 | Trichoderma spp |
| 4 | Oryza sativa | Rhizosphere | Soumousso | October-2018 | Trichoderma spp |
| 8 | Solanum melongena | Rhizosphere | Soumousso | October-2018 | Trichoderma spp |
| 2 | Ipomoea batatas | Rhizosphere | Soumousso | October-2018 | Aspergilus |
| 3 | Musa paradisiaca | Rhizosphere | Soumousso | October-2018 | Trichoderma spp |
| 1 | Portulaca grandiflora | Rhizosphere | Soumousso | October-2018 | Trichoderma spp |
| 1 | Hibiscus sabdariffa | Rhizosphere | Soumousso | October-2018 | Beauveria spp |
| 1 | Corchorus olitorius | Rhizosphere | Soumousso | October-2018 | Trichoderma spp |
| 3 | Cymbopogon citratus | Rhizosphere | Soumousso | October-2018 | Aspergilus |
| 1 | Acacia ataxacantha | Rhizosphere | Soumousso | October-2018 | Metarhizium spp |
| 1 | PsNDium guajava | Rhizosphere | Soumousso | October-2018 | Metarhizium spp |
| 1 | Phaseolus vulgaris | Rhizosphere | Soumousso | October-2018 | Fusarium spp |
| 6 | Annona squamosa | Rhizosphere | Soumousso | October-2018 | Fusarium spp |
| 2 | Calotropopis procera | Rhizosphere | Soumousso | October-2018 | Fusarium spp |
| 3 | Zea mays | Rhizosphere | Soumousso | October-2018 | Aspergilus |
| 1 | Abelmoschus esculentus | Rhizosphere | Soumousso | October-2018 | Beauveria spp |
| 4 | Oryza sativa ( Asian variety) | Rhizosphere | Soumousso | October-2018 | Aspergilus |
| 4 | Nauclea latifolia | Rhizosphere | Soumousso | October-2018 | Aspergilus |
| 9 | Green beans (Phaseolus vulgaris) | Rhizosphere | Soumousso | October-2018 | Aspergilus |
| 1 | Zea mays | Rhizosphere | Soumousso | October-2018 | Trichoderma spp |
| 3 | Sesamum indicum | Rhizosphere | Soumousso | October-2018 | Trichoderma spp |
| 1 | Vernonia colorata | Rhizosphere | Soumousso | October-2018 | ND |
| 1 | PsNDium guajava | Rhizosphere | Soumousso | October-2018 | Trichoderma spp |
| 3 | Nauclea latifolia | Rhizosphere | Soumousso | October-2018 | Trichoderma spp |
| 5 | Arachis hypogaea | Rhizosphere | Soumousso | October-2018 | Trichoderma spp |
| 5 | Parkia biglobosa | Rhizosphere | Soumousso | October-2018 | Trichoderma spp |
| 2 | Saba senegalensis | Rhizosphere | Soumousso | October-2018 | Trichoderma spp |
| 2 | Phaseolus vulgaris | Rhizosphere | Soumousso | October-2018 | Trichoderma spp |
| 4 | Oryza sativa ( Asian variety) | Rhizosphere | Soumousso | October-2018 | Trichoderma spp |
| 5 | Sorghum bicolor | Rhizosphere | Soumousso | October-2018 | Beauveria spp |
| 5 | Tamarindus indica | Rhizosphere | Soumousso | October-2018 | ND |
| 4 | Corchorus olitorius | Rhizosphere | Soumousso | October-2018 | Trichoderma spp |
| 3 | Ipomoea batatas | Rhizosphere | Soumousso | October-2018 | Trichoderma spp |
| 2 | Tectona grandis | Rhizosphere | Soumousso | October-2018 | ND |
| 1 | Vitellaria paradoxa | Rhizosphere | Soumousso | October-2018 | Metarhizium spp |
| 2 | mangifera indica | Rhizosphere | Soumousso | October-2018 | Fusarium spp |
| 2 | Phaseolus vulgaris L | Rhizosphere | Soumousso | October-2018 | ND |
| 3 | Lannea microcarpa | Rhizosphere | Soumousso | October-2018 | Aspergilus |
| 4 | Combretum paniculatum | Rhizosphere | Soumousso | October-2018 | Aspergilus |
| 2 | Saba senegalensis | Rhizosphere | Soumousso | October-2018 | Beauveria spp |
| 1 | Vernonia colorata | Rhizosphere | Soumousso | October-2018 | Aspergilus |
| 1 | Musa paradisiaca | Rhizosphere | Soumousso | October-2018 | Trichoderma spp |
| 2 | Green beans (Phaseolus vulgaris) | Rhizosphere | Soumousso | October-2018 | ND |
| 1 | Banana* (Tree) | Rhizosphere | Soumousso | October-2018 | Trichoderma spp |
| 1 | Zea mays | Rhizosphere | Soumousso | October-2018 | Trichoderma spp |
| 1 | Abelmoschus esculentus | Rhizosphere | Soumousso | October-2018 | ND |
| 2 | Oryza sativa ( Asian variety) | Rhizosphere | Soumousso | October-2018 | Trichoderma spp |
| 1 | Portulaca grandiflora | Rhizosphere | Soumousso | October-2018 | Trichoderma spp |
| 2 | Nauclea latifolia | Rhizosphere | Soumousso | October-2018 | Trichoderma spp |
| 2 | Green beans (Phaseolus vulgaris) | Rhizosphere | Soumousso | October-2018 | ND |
| 2 | Zea mays | Rhizosphere | Soumousso | October-2018 | Metarhizium spp |
| 1 | Sesamum indicum | Rhizosphere | Soumousso | October-2018 | Metarhizium spp |
| 2 | Vernonia colorata | Rhizosphere | Soumousso | October-2018 | Metarhizium spp |
| 3 | PsNDium guajava | Rhizosphere | Soumousso | October-2018 | Metarhizium spp |
| 2 | Nauclea latifolia | Rhizosphere | Soumousso | October-2018 | Metarhizium spp |
| 1 | Parkia biglobosa | Rhizosphere | Soumousso | October-2018 | Metarhizium spp |
| 1 | Saba senegalensis | Rhizosphere | Soumousso | October-2018 | Fusarium spp |
| 1 | Phaseolus vulgaris | Rhizosphere | Soumousso | October-2018 | Fusarium spp |
| 1 | Oryza sativa ( Asian variety) | Rhizosphere | Soumousso | October-2018 | Fusarium spp |
| 1 | Sorghum bicolor | Rhizosphere | Soumousso | October-2018 | Fusarium spp |
| 1 | Combretum paniculatum | Rhizosphere | Soumousso | October-2018 | Fusarium spp |
| 2 | Saba senegalensis | Rhizosphere | Soumousso | October-2018 | Fusarium spp |
| 2 | Vernonia colorata | Rhizosphere | Soumousso | October-2018 | Fusarium spp |
| 1 | Musa paradisiaca | Rhizosphere | Soumousso | October-2018 | Fusarium spp |
| 1 | Green beans (Phaseolus vulgaris) | Rhizosphere | Soumousso | October-2018 | Fusarium spp |
| 3 | Banana* (Tree) | Rhizosphere | Soumousso | October-2018 | Fusarium spp |
| 1 | Zea mays | Rhizosphere | Soumousso | October-2018 | Beauveria spp |
| 1 | Abelmoschus esculentus | Rhizosphere | Soumousso | October-2018 | Aspergilus |
| 2 | Phragmites australis | Rhizosphere | Soumousso | October-2018 | Aspergilus |
| 2 | Khaya senegalensis | Rhizosphere | Soumousso | October-2018 | Aspergilus |
| 2 | Azadirachta indica | Rhizosphere | Soumousso | October-2018 | Aspergilus |
| 1 | Sclerocarya birrea | Rhizosphere | Soumousso | October-2018 | Trichoderma spp |
| 2 | Thevetia neriifolia | Rhizosphere | Soumousso | October-2018 | Trichoderma spp |
| 1 | Adansonia digitata | Rhizosphere | Soumousso | October-2018 | Trichoderma spp |
| 2 | Bombax costatum | Rhizosphere | Soumousso | October-2018 | Beauveria spp |
| 2 | Zea mays | Rhizosphere | Soumousso | October-2018 | Trichoderma spp |
| 1 | Sorghum bicolor | Rhizosphere | Soumousso | October-2018 | Trichoderma spp |
| 2 | Sesamum indicum | Rhizosphere | Soumousso | October-2018 | Trichoderma spp |
| 1 | Nicotiana tabacum | Rhizosphere | Soumousso | October-2018 | Trichoderma spp |
| 1 | Tectona grandis | Rhizosphere | Soumousso | October-2018 | Trichoderma spp |
| 5 | Vitellaria paradoxa | Rhizosphere | Soumousso | October-2018 | Aspergilus |
| 1 | mangifera indica | Rhizosphere | Soumousso | October-2018 | Aspergilus |
| 1 | Phaseolus vulgaris L | Rhizosphere | Soumousso | October-2018 | Aspergilus |
| 2 | Lannea microcarpa | Rhizosphere | Soumousso | October-2018 | Aspergilus |
| 1 | Balanites aegytiaca | Rhizosphere | Soumousso | October-2018 | Aspergilus |
| 2 | Saba senegalensis | Rhizosphere | Soumousso | October-2018 | Aspergilus |
| 1 | Nauclea latifolia | Rhizosphere | Soumousso | October-2018 | Aspergilus |
| 1 | wild grass | Rhizosphere | Soumousso | October-2018 | Aspergilus |
| 1 | Parkia biglobosa | Rhizosphere | Soumousso | October-2018 | Aspergilus |
| 3 | Piliostigma thonningii | Rhizosphere | Soumousso | October-2018 | Aspergilus |
| 1 | Cassia siamea | Rhizosphere | Soumousso | October-2018 | ND |
| 2 | Phragmites australis | Rhizosphere | Soumousso | October-2018 | Aspergilus |
| 1 | Khaya senegalensis | Rhizosphere | Soumousso | October-2018 | Aspergilus |
| 2 | Azadirachta indica | Rhizosphere | Soumousso | October-2018 | Aspergilus |
| 1 | Sclerocarya birrea | Rhizosphere | Soumousso | October-2018 | Aspergilus |
| 2 | Thevetia neriifolia | Rhizosphere | Soumousso | October-2018 | Aspergilus |
| 2 | Adansonia digitata | Rhizosphere | Soumousso | October-2018 | Aspergilus |
| 1 | Bombax costatum | Rhizosphere | Soumousso | October-2018 | Aspergilus |
| 4 | Zea mays | Rhizosphere | Soumousso | October-2018 | Aspergilus |
| 2 | Sorghum bicolor | Rhizosphere | Soumousso | October-2018 | ND |
| 2 | Sesamum indicum | Rhizosphere | Soumousso | October-2018 | Aspergilus |
| 1 | Nicotiana tabacum | Rhizosphere | Soumousso | October-2018 | Aspergilus |
| 1 | Tectona grandis | Rhizosphere | Soumousso | October-2018 | Trichoderma spp |
| 3 | Vitellaria paradoxa | Rhizosphere | Soumousso | October-2018 | Trichoderma spp |
| 1 | mangifera indica | Rhizosphere | Soumousso | October-2018 | Trichoderma spp |
| 2 | Phaseolus vulgaris L | Rhizosphere | Soumousso | October-2018 | Trichoderma spp |
| 1 | Lannea microcarpa | Rhizosphere | Soumousso | October-2018 | Trichoderma spp |
| 3 | Balanites aegytiaca | Rhizosphere | Soumousso | October-2018 | Trichoderma spp |
| 2 | Saba senegalensis | Rhizosphere | Soumousso | October-2018 | Trichoderma spp |
| 2 | Nauclea latifolia | Rhizosphere | Soumousso | October-2018 | Trichoderma spp |
| 1 | wild grass | Rhizosphere | Soumousso | October-2018 | Trichoderma spp |
| 1 | Parkia biglobosa | Rhizosphere | Soumousso | October-2018 | Trichoderma spp |
| 2 | Piliostigma thonningii | Rhizosphere | Soumousso | October-2018 | Trichoderma spp |
| 2 | Cassia siamea | Rhizosphere | Soumousso | October-2018 | Trichoderma spp |
| 2 | Phragmites australis | Rhizosphere | Soumousso | October-2018 | Trichoderma spp |
| 1 | Khaya senegalensis | Rhizosphere | Soumousso | October-2018 | Trichoderma spp |
| 3 | Azadirachta indica | Rhizosphere | Soumousso | October-2018 | Trichoderma spp |
| 1 | Sclerocarya birrea | Rhizosphere | Soumousso | October-2018 | Trichoderma spp |
| 2 | Thevetia neriifolia | Rhizosphere | Soumousso | October-2018 | ND |
| 1 | Adansonia digitata | Rhizosphere | Soumousso | October-2018 | Trichoderma spp |
| 1 | Bombax costatum | Rhizosphere | Soumousso | October-2018 | Trichoderma spp |
| 1 | Zea mays | Rhizosphere | Soumousso | October-2018 | Trichoderma spp |
| 1 | Sorghum bicolor | Rhizosphere | Soumousso | October-2018 | Trichoderma spp |
| 1 | Sesamum indicum | Rhizosphere | Soumousso | October-2018 | Trichoderma spp |
| 1 | Nicotiana tabacum | Rhizosphere | Soumousso | October-2018 | Beauveria spp |
| 4 | Tectona grandis | Rhizosphere | Soumousso | October-2018 | Beauveria spp |
| 1 | Vitellaria paradoxa | Rhizosphere | Soumousso | October-2018 | Beauveria spp |
| 1 | mangifera indica | Rhizosphere | Soumousso | October-2018 | Beauveria spp |
| 2 | Phaseolus vulgaris L | Rhizosphere | Soumousso | October-2018 | Beauveria spp |
| 3 | Lannea microcarpa | Rhizosphere | Soumousso | October-2018 | Beauveria spp |
| 1 | Balanites aegytiaca | Rhizosphere | Soumousso | October-2018 | Beauveria spp |
| 2 | Saba senegalensis | Rhizosphere | Soumousso | October-2018 | Beauveria spp |
| 1 | Nauclea latifolia | Rhizosphere | Soumousso | October-2018 | Beauveria spp |
| 2 | wild grass | Rhizosphere | Soumousso | October-2018 | Beauveria spp |
| 3 | Parkia biglobosa | Rhizosphere | Soumousso | October-2018 | Beauveria spp |
| 1 | Piliostigma thonningii | Rhizosphere | Soumousso | October-2018 | Beauveria spp |
| 1 | Cassia siamea | Rhizosphere | Soumousso | October-2018 | Beauveria spp |
| 1 | Phragmites australis | Rhizosphere | Soumousso | October-2018 | Beauveria spp |
| 3 | Khaya senegalensis | Rhizosphere | Soumousso | October-2018 | Trichoderma spp |
| 3 | Azadirachta indica | Rhizosphere | Soumousso | October-2018 | Trichoderma spp |
| 1 | Sclerocarya birrea | Rhizosphere | Soumousso | October-2018 | Metarhizium spp |
| 2 | Thevetia neriifolia | Rhizosphere | Soumousso | October-2018 | Metarhizium spp |
| 4 | Adansonia digitata | Rhizosphere | Soumousso | October-2018 | Metarhizium spp |
| 1 | Bombax costatum | Rhizosphere | Soumousso | October-2018 | Metarhizium spp |
| 2 | Zea mays | Rhizosphere | Soumousso | October-2018 | Metarhizium spp |
| 5 | Sorghum bicolor | Rhizosphere | Soumousso | October-2018 | ND |
| 1 | Sesamum indicum | Rhizosphere | Soumousso | October-2018 | Metarhizium spp |
| 1 | Nicotiana tabacum | Rhizosphere | Soumousso | October-2018 | ND |
| 1 | Tectona grandis | Rhizosphere | Soumousso | October-2018 | Fusarium spp |
| 1 | Vitellaria paradoxa | Rhizosphere | Soumousso | October-2018 | Aspergilus |
| 1 | mangifera indica | Rhizosphere | Soumousso | October-2018 | Aspergilus |
| 1 | Phaseolus vulgaris L | Rhizosphere | Soumousso | October-2018 | Aspergilus |
| 1 | Lannea microcarpa | Rhizosphere | Soumousso | October-2018 | Aspergilus |
| 1 | Balanites aegytiaca | Rhizosphere | Soumousso | October-2018 | Aspergilus |
| 1 | Saba senegalensis | Rhizosphere | Soumousso | October-2018 | Trichoderma spp |
| 2 | Nauclea latifolia | Rhizosphere | Soumousso | October-2018 | Trichoderma spp |
| 1 | wild grass | Rhizosphere | Soumousso | October-2018 | Trichoderma spp |
| 1 | Parkia biglobosa | Rhizosphere | Soumousso | October-2018 | Trichoderma spp |
| 1 | Piliostigma thonningii | Rhizosphere | Soumousso | October-2018 | Trichoderma spp |
| 1 | Cassia siamea | Rhizosphere | Soumousso | October-2018 | Trichoderma spp |
| 1 | Phragmites australis | Rhizosphere | Soumousso | October-2018 | Trichoderma spp |
| 2 | Khaya senegalensis | Rhizosphere | Soumousso | October-2018 | Beauveria spp |
| 2 | Azadirachta indica | Rhizosphere | Soumousso | October-2018 | Trichoderma spp |
| 1 | Sclerocarya birrea | Rhizosphere | Soumousso | October-2018 | Fusarium spp |
| 1 | Thevetia neriifolia | Rhizosphere | Soumousso | October-2018 | Fusarium spp |
| 1 | Adansonia digitata | Rhizosphere | Soumousso | October-2018 | Fusarium spp |
| 2 | Bombax costatum | Rhizosphere | Soumousso | October-2018 | Fusarium spp |
| 2 | Zea mays | Rhizosphere | Soumousso | October-2018 | Fusarium spp |
| 1 | Sorghum bicolor | Rhizosphere | Soumousso | October-2018 | Fusarium spp |
| 1 | Sesamum indicum | Rhizosphere | Soumousso | October-2018 | Fusarium spp |
| 1 | Nicotiana tabacum | Rhizosphere | Soumousso | October-2018 | Fusarium spp |
| 2 | Tectona grandis | Rhizosphere | Soumousso | October-2018 | Aspergilus |
| 1 | Vitellaria paradoxa | Rhizosphere | Soumousso | October-2018 | Beauveria spp |
| 1 | mangifera indica | Rhizosphere | Soumousso | October-2018 | Aspergilus |
| 1 | Phaseolus vulgaris L | Rhizosphere | Soumousso | October-2018 | Aspergilus |
| 2 | Lannea microcarpa | Rhizosphere | Soumousso | October-2018 | Aspergilus |
| 1 | Balanites aegytiaca | Rhizosphere | Soumousso | October-2018 | ND |
| 1 | Saba senegalensis | Rhizosphere | Soumousso | October-2018 | Trichoderma spp |
| 1 | Nauclea latifolia | Rhizosphere | Soumousso | October-2018 | Trichoderma spp |
| 1 | wild grass | Rhizosphere | Soumousso | October-2018 | ND |
| 1 | Parkia biglobosa | Rhizosphere | Soumousso | October-2018 | Trichoderma spp |
| 2 | Piliostigma thonningii | Rhizosphere | Soumousso | October-2018 | Trichoderma spp |
| 1 | Cassia siamea | Rhizosphere | Soumousso | October-2018 | Trichoderma spp |
| 1 | Phragmites australis | Rhizosphere | Soumousso | October-2018 | Trichoderma spp |
| 1 | Khaya senegalensis | Rhizosphere | Soumousso | October-2018 | Trichoderma spp |
| 1 | Azadirachta indica | Rhizosphere | Soumousso | October-2018 | Aspergilus |
| 1 | Sclerocarya birrea | Rhizosphere | Soumousso | October-2018 | Aspergilus |
| 1 | Thevetia neriifolia | Rhizosphere | Soumousso | October-2018 | Aspergilus |
| 2 | Adansonia digitata | Rhizosphere | Soumousso | October-2018 | ND |
| 4 | Bombax costatum | Rhizosphere | Soumousso | October-2018 | Aspergilus |
| 1 | Zea mays | Rhizosphere | Soumousso | October-2018 | Aspergilus |
| 1 | Sorghum bicolor | Rhizosphere | Soumousso | October-2018 | Fusarium spp |
| 2 | Sesamum indicum | Rhizosphere | Soumousso | October-2018 | Aspergilus |
| 1 | Nicotiana tabacum | Rhizosphere | Soumousso | October-2018 | Aspergilus |
| 1 | Tectona grandis | Rhizosphere | Soumousso | October-2018 | Aspergilus |
| 2 | Vitellaria paradoxa | Rhizosphere | Soumousso | October-2018 | Aspergilus |
| 1 | mangifera indica | Rhizosphere | Soumousso | October-2018 | Aspergilus |
| 1 | Phaseolus vulgaris L | Rhizosphere | Soumousso | October-2018 | Trichoderma spp |
| 1 | Lannea microcarpa | Rhizosphere | Soumousso | October-2018 | Trichoderma spp |
| 1 | Balanites aegytiaca | Rhizosphere | Soumousso | October-2018 | Trichoderma spp |
| 2 | Saba senegalensis | Rhizosphere | Soumousso | October-2018 | Trichoderma spp |
| 1 | Nauclea latifolia | Rhizosphere | Soumousso | October-2018 | Beauveria spp |
| 1 | wild grass | Rhizosphere | Soumousso | October-2018 | ND |
| 3 | Parkia biglobosa | Rhizosphere | Soumousso | October-2018 | Beauveria spp |
| 1 | Piliostigma thonningii | Rhizosphere | Soumousso | October-2018 | Beauveria spp |
| 1 | Cassia siamea | Rhizosphere | Soumousso | October-2018 | Beauveria spp |
| 1 | Phragmites australis | Rhizosphere | Soumousso | October-2018 | ND |
| 2 | Khaya senegalensis | Rhizosphere | Soumousso | October-2018 | Beauveria spp |
| 1 | Azadirachta indica | Rhizosphere | Soumousso | October-2018 | Beauveria spp |
| 1 | Sclerocarya birrea | Rhizosphere | Soumousso | October-2018 | Beauveria spp |
| 2 | Thevetia neriifolia | Rhizosphere | Soumousso | October-2018 | Beauveria spp |
| 1 | Adansonia digitata | Rhizosphere | Soumousso | October-2018 | ND |
| 1 | Bombax costatum | Rhizosphere | Soumousso | October-2018 | Trichoderma spp |
| 2 | Zea mays | Rhizosphere | Soumousso | October-2018 | Trichoderma spp |
| 1 | Sorghum bicolor | Rhizosphere | Soumousso | October-2018 | Trichoderma spp |
| 1 | Sesamum indicum | Rhizosphere | Soumousso | October-2018 | Trichoderma spp |
| 3 | Nicotiana tabacum | Rhizosphere | Soumousso | October-2018 | Trichoderma spp |
| 1 | Tectona grandis | Rhizosphere | Soumousso | October-2018 | Fusarium spp |
| 1 | Vitellaria paradoxa | Rhizosphere | Soumousso | October-2018 | Aspergilus |
| 2 | mangifera indica | Rhizosphere | Soumousso | October-2018 | Aspergilus |
| 1 | Phaseolus vulgaris L | Rhizosphere | Soumousso | October-2018 | Aspergilus |
| 2 | Lannea microcarpa | Rhizosphere | Soumousso | October-2018 | Aspergilus |
| 2 | Balanites aegytiaca | Rhizosphere | Soumousso | October-2018 | Aspergilus |
| 1 | Saba senegalensis | Rhizosphere | Soumousso | October-2018 | Trichoderma spp |
| 2 | Nauclea latifolia | Rhizosphere | Soumousso | October-2018 | Trichoderma spp |
| 4 | wild grass | Rhizosphere | Soumousso | October-2018 | Trichoderma spp |
| 2 | Parkia biglobosa | Rhizosphere | Soumousso | October-2018 | Trichoderma spp |
| 4 | Piliostigma thonningii | Rhizosphere | Soumousso | October-2018 | Trichoderma spp |
| 3 | Cassia siamea | Rhizosphere | Soumousso | October-2018 | Trichoderma spp |
| 1 | Phragmites australis | Rhizosphere | Soumousso | October-2018 | Trichoderma spp |
| 1 | Khaya senegalensis | Rhizosphere | Soumousso | October-2018 | ND |
| 1 | Azadirachta indica | Rhizosphere | Soumousso | October-2018 | Trichoderma spp |
| 2 | Sclerocarya birrea | Rhizosphere | Soumousso | October-2018 | Metarhizium spp |
| 1 | Thevetia neriifolia | Rhizosphere | Soumousso | October-2018 | Metarhizium spp |
| 2 | Adansonia digitata | Rhizosphere | Soumousso | October-2018 | Fusarium spp |
| 1 | Bombax costatum | Rhizosphere | Soumousso | October-2018 | Aspergilus |
| 1 | Zea mays | Rhizosphere | Soumousso | October-2018 | Beauveria spp |
| 2 | Sorghum bicolor | Rhizosphere | Soumousso | October-2018 | ND |
| 2 | Sesamum indicum | Rhizosphere | Soumousso | October-2018 | Beauveria spp |
| 1 | Nicotiana tabacum | Rhizosphere | Soumousso | October-2018 | Beauveria spp |
| 1 | Tectona grandis | Rhizosphere | Soumousso | October-2018 | Beauveria spp |
| 2 | Vitellaria paradoxa | Rhizosphere | Soumousso | October-2018 | Beauveria spp |
| 1 | mangifera indica | Rhizosphere | Soumousso | October-2018 | Beauveria spp |
| 1 | Phaseolus vulgaris L | Rhizosphere | Soumousso | October-2018 | ND |
| 1 | Lannea microcarpa | Rhizosphere | Soumousso | October-2018 | Beauveria spp |
| 3 | Balanites aegytiaca | Rhizosphere | Soumousso | October-2018 | Trichoderma spp |
| 1 | Saba senegalensis | Rhizosphere | Soumousso | October-2018 | Trichoderma spp |
| 1 | Nauclea latifolia | Rhizosphere | Soumousso | October-2018 | Trichoderma spp |
| 1 | wild grass | Rhizosphere | Soumousso | October-2018 | Trichoderma spp |
| 2 | Parkia biglobosa | Rhizosphere | Soumousso | October-2018 | Fusarium spp |
| 1 | Piliostigma thonningii | Rhizosphere | Soumousso | October-2018 | Fusarium spp |
| 4 | Cassia siamea | Rhizosphere | Soumousso | October-2018 | Fusarium spp |
| 1 | Phragmites australis | Rhizosphere | Soumousso | October-2018 | Fusarium spp |
| 2 | Khaya senegalensis | Rhizosphere | Soumousso | October-2018 | Fusarium spp |
| 1 | Azadirachta indica | Rhizosphere | Soumousso | October-2018 | Fusarium spp |
| 2 | Sclerocarya birrea | Rhizosphere | Soumousso | October-2018 | Fusarium spp |
| 1 | Thevetia neriifolia | Rhizosphere | Soumousso | October-2018 | Fusarium spp |
| 1 | Adansonia digitata | Rhizosphere | Soumousso | October-2018 | Fusarium spp |
| 1 | Bombax costatum | Rhizosphere | Soumousso | October-2018 | Aspergilus |
| 1 | Zea mays | Rhizosphere | Soumousso | October-2018 | Aspergilus |
| 1 | Sorghum bicolor | Rhizosphere | Soumousso | October-2018 | Aspergilus |
| 1 | Sesamum indicum | Rhizosphere | Soumousso | October-2018 | Aspergilus |
| 3 | Nicotiana tabacum | Rhizosphere | Soumousso | October-2018 | Aspergilus |
| 1 | Tectona grandis | Rhizosphere | Soumousso | October-2018 | Trichoderma spp |
| 1 | Vitellaria paradoxa | Rhizosphere | Soumousso | October-2018 | Trichoderma spp |
| 2 | mangifera indica | Rhizosphere | Soumousso | October-2018 | Trichoderma spp |
| 1 | Phaseolus vulgaris L | Rhizosphere | Soumousso | October-2018 | Trichoderma spp |
| 1 | Lannea microcarpa | Rhizosphere | Soumousso | October-2018 | Trichoderma spp |
| 3 | Balanites aegytiaca | Rhizosphere | Soumousso | October-2018 | Trichoderma spp |
| 1 | Saba senegalensis | Rhizosphere | Soumousso | October-2018 | Beauveria spp |
| 1 | Nauclea latifolia | Rhizosphere | Soumousso | October-2018 | Beauveria spp |
| 1 | wild grass | Rhizosphere | Soumousso | October-2018 | Beauveria spp |
| 2 | Parkia biglobosa | Rhizosphere | Soumousso | October-2018 | Beauveria spp |
| 1 | Piliostigma thonningii | Rhizosphere | Soumousso | October-2018 | Beauveria spp |
| 1 | Cassia siamea | Rhizosphere | Soumousso | October-2018 | Beauveria spp |
| 2 | Phragmites australis | Rhizosphere | Soumousso | October-2018 | Aspergilus |
| 1 | Khaya senegalensis | Rhizosphere | Soumousso | October-2018 | Aspergilus |
| 1 | Azadirachta indica | Rhizosphere | Soumousso | October-2018 | Aspergilus |
| 1 | Sclerocarya birrea | Rhizosphere | Soumousso | October-2018 | Aspergilus |
| 1 | Thevetia neriifolia | Rhizosphere | Soumousso | October-2018 | Fusarium spp |
| 2 | Adansonia digitata | Rhizosphere | Soumousso | October-2018 | ND |
| 1 | Bombax costatum | Rhizosphere | Soumousso | October-2018 | Aspergilus |
| 2 | Zea mays | Rhizosphere | Soumousso | October-2018 | Aspergilus |
| 1 | Sorghum bicolor | Rhizosphere | Soumousso | October-2018 | Aspergilus |
| 1 | Sesamum indicum | Rhizosphere | Soumousso | October-2018 | Aspergilus |
| 1 | Nicotiana tabacum | Rhizosphere | Soumousso | October-2018 | Trichoderma spp |
| 1 | Tectona grandis | Rhizosphere | Soumousso | October-2018 | Beauveria spp |
| 1 | Vitellaria paradoxa | Rhizosphere | Soumousso | October-2018 | Beauveria spp |
| 2 | mangifera indica | Rhizosphere | Soumousso | October-2018 | Beauveria spp |
| 1 | Phaseolus vulgaris L | Rhizosphere | Soumousso | October-2018 | Beauveria spp |
| 2 | Lannea microcarpa | Rhizosphere | Soumousso | October-2018 | Beauveria spp |
| 1 | Balanites aegytiaca | Rhizosphere | Soumousso | October-2018 | Beauveria spp |
| 2 | Saba senegalensis | Rhizosphere | Soumousso | October-2018 | Beauveria spp |
| 1 | Nauclea latifolia | Rhizosphere | Soumousso | October-2018 | Beauveria spp |
| 2 | wild grass | Rhizosphere | Soumousso | October-2018 | Beauveria spp |
| 1 | Parkia biglobosa | Rhizosphere | Soumousso | October-2018 | Trichoderma spp |
| 1 | Piliostigma thonningii | Rhizosphere | Soumousso | October-2018 | Trichoderma spp |
| 2 | Cassia siamea | Rhizosphere | Soumousso | October-2018 | Trichoderma spp |
| 1 | Senna siamea | Rhizosphere | Soumousso | October-2018 | Trichoderma spp |
| 1 | Khaya senegalensis | Rhizosphere | Soumousso | October-2018 | Trichoderma spp |
| 2 | Azadirachta indica | Rhizosphere | Soumousso | October-2018 | Trichoderma spp |
| 1 | Sclerocarya birrea | Rhizosphere | Soumousso | October-2018 | Fusarium spp |
| 2 | Thevetia neriifolia | Rhizosphere | Soumousso | October-2018 | Aspergilus |
| 1 | Adansonia digitata | Rhizosphere | Soumousso | October-2018 | Aspergilus |
| 1 | Bombax costatum | Rhizosphere | Soumousso | October-2018 | Aspergilus |
| 2 | Zea mays | Rhizosphere | Soumousso | October-2018 | Aspergilus |
| 1 | Sorghum bicolor | Rhizosphere | Soumousso | October-2018 | Aspergilus |
| 1 | Sesamum indicum | Rhizosphere | Soumousso | October-2018 | Trichoderma spp |
| 1 | Nicotiana tabacum | Rhizosphere | Soumousso | October-2018 | Trichoderma spp |
| 2 | Musa paradisiaca | Rhizosphere | Soumousso | October-2018 | Trichoderma spp |
| 1 | Portulaca grandiflora | Rhizosphere | Soumousso | October-2018 | Beauveria spp |
| 2 | Hibiscus sabdariffa | Rhizosphere | Soumousso | October-2018 | Beauveria spp |
| 1 | Corchorus olitorius | Rhizosphere | Soumousso | October-2018 | Beauveria spp |
| 1 | Cymbopogon citratus | Rhizosphere | Soumousso | October-2018 | Beauveria spp |
| 2 | Acacia ataxacantha | Rhizosphere | Soumousso | October-2018 | Beauveria spp |
| 1 | PsNDium guajava | Rhizosphere | Soumousso | October-2018 | Beauveria spp |
| 3 | Phaseolus vulgaris | Rhizosphere | Soumousso | October-2018 | Beauveria spp |
| 1 | Annona squamosa | Rhizosphere | Soumousso | October-2018 | Beauveria spp |
| 1 | Calotropopis procera | Rhizosphere | Soumousso | October-2018 | Metarhizium spp |
| 1 | Lannea microcarpa | Rhizosphere | Soumousso | October-2018 | Metarhizium spp |
| 1 | Sesamum indicum | Rhizosphere | Soumousso | October-2018 | Metarhizium spp |
| 2 | Vigna subterranea | Rhizosphere | Soumousso | October-2018 | Metarhizium spp |
| 1 | Vitellaria paradoxa | Rhizosphere | Soumousso | October-2018 | Metarhizium spp |
| 1 | Solanum lycopersicum | Rhizosphere | Soumousso | October-2018 | Metarhizium spp |
| 1 | Thevetia neriifolia | Rhizosphere | Soumousso | October-2018 | Metarhizium spp |
| 1 | Bombax costatum | Rhizosphere | Soumousso | October-2018 | Fusarium spp |
| 1 | Lagenaria siceraria | Rhizosphere | Soumousso | October-2018 | Aspergilus |
| 1 | Jatropha curcas | Rhizosphere | Soumousso | October-2018 | ND |
| 1 | Anopheles gambiae sl | Rhizosphere | Soumousso | October-2018 | Aspergilus |
| 1 | Culex sp | Rhizosphere | Soumousso | October-2018 | Aspergilus |
| 3 | Culex sp | Rhizosphere | Soumousso | October-2018 | Aspergilus |
| 1 | Culex sp | Rhizosphere | Soumousso | October-2018 | Trichoderma spp |
| 1 | Anopheles pharoensis | Rhizosphere | Soumousso | October-2018 | Trichoderma spp |
| 4 | Anopheles pharoensis | Rhizosphere | Soumousso | October-2018 | Trichoderma spp |
| 1 | Anopheles pharoensis | Rhizosphere | Soumousso | October-2018 | Trichoderma spp |
| 2 | Anopheles gambiae sl | Rhizosphere | Soumousso | October-2018 | Trichoderma spp |
| 1 | Anopheles gambiae sl | Rhizosphere | Soumousso | October-2018 | Trichoderma spp |
| 1 | Anopheles gambiae sl | Rhizosphere | Soumousso | October-2018 | Trichoderma spp |
| 1 | Culex sp | Rhizosphere | Soumousso | October-2018 | Trichoderma spp |
| 2 | Culex sp | Rhizosphere | Soumousso | October-2018 | Trichoderma spp |
| 1 | Culex sp | Rhizosphere | Soumousso | October-2018 | Metarhizium spp |
| 1 | Anopheles pharoensis | Rhizosphere | Soumousso | October-2018 | Fusarium spp |
| 1 | Anopheles pharoensis | Rhizosphere | Soumousso | October-2018 | Fusarium spp |
| 1 | Anopheles pharoensis | Rhizosphere | Soumousso | October-2018 | Fusarium spp |
| 1 | Anopheles gambiae sl | Rhizosphere | Soumousso | October-2018 | Fusarium spp |
| 1 | Anopheles gambiae sl | Rhizosphere | Soumousso | October-2018 | Fusarium spp |
| 2 | Anopheles gambiae sl | Rhizosphere | Soumousso | October-2018 | Beauveria spp |
| 3 | Culex sp | Rhizosphere | Soumousso | October-2018 | Beauveria spp |
| 1 | Culex sp | Rhizosphere | Soumousso | October-2018 | Beauveria spp |
| 1 | Culex sp | Rhizosphere | Soumousso | October-2018 | Beauveria spp |
| 1 | Anopheles pharoensis | Rhizosphere | Soumousso | October-2018 | Beauveria spp |
| 2 | Anopheles pharoensis | Rhizosphere | Soumousso | October-2018 | Beauveria spp |
| 1 | Anopheles pharoensis | Rhizosphere | Soumousso | October-2018 | Beauveria spp |
| 1 | Anopheles gambiae sl | Rhizosphere | Soumousso | October-2018 | Beauveria spp |
| 2 | Anopheles gambiae sl | Rhizosphere | Soumousso | October-2018 | Beauveria spp |
| 2 | Anopheles gambiae sl | Rhizosphere | Soumousso | October-2018 | Beauveria spp |
| 1 | Anopheles gambiae sl | Rhizosphere | Soumousso | October-2018 | Beauveria spp |
| 1 | Anopheles gambiae sl | Rhizosphere | Soumousso | October-2018 | Beauveria spp |
| 1 | Lannea microcarpa | Rhizosphere | Soumousso | October-2018 | Beauveria spp |
| 1 | Sesamum indicum | Rhizosphere | Soumousso | October-2018 | Trichoderma spp |
| 1 | Vigna subterranea | Rhizosphere | Soumousso | October-2018 | Trichoderma spp |
| 1 | Vitellaria paradoxa | Rhizosphere | Soumousso | October-2018 | Trichoderma spp |
| 1 | Solanum lycopersicum | Rhizosphere | Soumousso | October-2018 | Fusarium spp |
| 1 | Thevetia neriifolia | Rhizosphere | Soumousso | October-2018 | Fusarium spp |
| 1 | Bombax costatum | Rhizosphere | Soumousso | October-2018 | Fusarium spp |
| 1 | Lagenaria siceraria | Rhizosphere | Soumousso | October-2018 | Fusarium spp |
| 1 | Jatropha curcas | Rhizosphere | Soumousso | October-2018 | Fusarium spp |
| 1 | Hibiscus rosa-sinensis | Rhizosphere | Soumousso | October-2018 | Fusarium spp |
| 1 | Balanites aegyptiaca | Rhizosphere | Soumousso | October-2018 | Fusarium spp |
| 1 | Zea mays | Rhizosphere | Soumousso | October-2018 | Fusarium spp |
| 1 | Nicotiana tabacum | Rhizosphere | Soumousso | October-2018 | Fusarium spp |
| 1 | Capsicum frutescens | Rhizosphere | Soumousso | October-2018 | Fusarium spp |
| 1 | Sorghum bicolor | Rhizosphere | Soumousso | October-2018 | Fusarium spp |
| 1 | Dioscorea alata | Rhizosphere | Soumousso | October-2018 | Aspergilus |
| 1 | Gossypium barbadense | Rhizosphere | Soumousso | October-2018 | Aspergilus |
| 1 | Vitex donania | Rhizosphere | Soumousso | October-2018 | Aspergilus |
| 1 | Arachis hypogaea | Rhizosphere | Soumousso | October-2018 | Aspergilus |
| 1 | Anopheles gambiae sl | Rhizosphere | Soumousso | October-2018 | Fusarium spp |
| 1 | Anopheles gambiae sl | Rhizosphere | Soumousso | October-2018 | Aspergilus |
| 1 | Anopheles gambiae sl | Rhizosphere | Soumousso | October-2018 | Beauveria spp |
| 1 | Culex sp | Rhizosphere | Soumousso | October-2018 | Beauveria spp |
| 1 | Culex sp | Rhizosphere | Soumousso | October-2018 | Beauveria spp |
| 1 | Culex sp | Rhizosphere | Soumousso | October-2018 | ND |
| 1 | Anopheles gambiae sl | Rhizosphere | Soumousso | October-2018 | Beauveria spp |
| 1 | Anopheles gambiae sl | Rhizosphere | Soumousso | October-2018 | Beauveria spp |
| 1 | Anopheles gambiae sl | Rhizosphere | Soumousso | October-2018 | Beauveria spp |
| 2 | Vitellaria paradoxa | Rhizosphere | Soumousso | October-2018 | Beauveria spp |
| 2 | Solanum lycopersicum | Rhizosphere | Soumousso | October-2018 | Beauveria spp |
| 1 | Thevetia neriifolia | Rhizosphere | Soumousso | October-2018 | Trichoderma spp |
| 2 | Bombax costatum | Rhizosphere | Soumousso | October-2018 | Trichoderma spp |
| 1 | Lagenaria siceraria | Rhizosphere | Soumousso | October-2018 | Trichoderma spp |
| 2 | Jatropha curcas | Rhizosphere | Soumousso | October-2018 | Trichoderma spp |
| 2 | Hibiscus rosa-sinensis | Rhizosphere | Soumousso | October-2018 | Aspergilus |
| 1 | Balanites aegyptiaca | Rhizosphere | Soumousso | October-2018 | Aspergilus |
| 1 | Zea mays | Rhizosphere | Soumousso | October-2018 | Aspergilus |
| 1 | Nicotiana tabacum | Rhizosphere | Soumousso | October-2018 | Aspergilus |
| 1 | Capsicum frutescens | Rhizosphere | Soumousso | October-2018 | Beauveria spp |
| 2 | Sorghum bicolor | Rhizosphere | Soumousso | October-2018 | Beauveria spp |
| 2 | Dioscorea alata | Rhizosphere | Soumousso | October-2018 | Beauveria spp |
| 1 | Gossypium barbadense | Rhizosphere | Soumousso | October-2018 | Beauveria spp |
| 1 | Vitex donania | Rhizosphere | Soumousso | October-2018 | Beauveria spp |
| 2 | Arachis hypogaea | Rhizosphere | Soumousso | October-2018 | Beauveria spp |
| 1 | Oryza sativa | Rhizosphere | Soumousso | October-2018 | Beauveria spp |
| 2 | Solanum melongena | Rhizosphere | Soumousso | October-2018 | Beauveria spp |
| 2 | Ipomoea batatas | Rhizosphere | Soumousso | October-2018 | Beauveria spp |
| 1 | Musa paradisiaca | Rhizosphere | Soumousso | October-2018 | Beauveria spp |
| 1 | Portulaca grandiflora | Rhizosphere | Soumousso | October-2018 | Beauveria spp |
| 1 | Hibiscus sabdariffa | Rhizosphere | Soumousso | October-2018 | Trichoderma spp |
| 1 | Corchorus olitorius | Rhizosphere | Soumousso | October-2018 | Trichoderma spp |
| 1 | Cymbopogon citratus | Rhizosphere | Soumousso | October-2018 | Trichoderma spp |
| 2 | Acacia ataxacantha | Rhizosphere | Soumousso | October-2018 | Trichoderma spp |
| 1 | PsNDium guajava | Rhizosphere | Soumousso | October-2018 | Trichoderma spp |
| 1 | Phaseolus vulgaris | Rhizosphere | Soumousso | October-2018 | Trichoderma spp |
| 1 | Annona squamosa | Rhizosphere | Soumousso | October-2018 | Trichoderma spp |
| 2 | Calotropopis procera | Rhizosphere | Soumousso | October-2018 | Trichoderma spp |
| 3 | Lannea microcarpa | Rhizosphere | Soumousso | October-2018 | Trichoderma spp |
| 1 | Sesamum indicum | Rhizosphere | Soumousso | October-2018 | Trichoderma spp |
| 1 | Vigna subterranea | Rhizosphere | Soumousso | October-2018 | Trichoderma spp |
| 2 | Vitellaria paradoxa | Rhizosphere | Soumousso | October-2018 | Beauveria spp |
| 2 | Solanum lycopersicum | Rhizosphere | Soumousso | October-2018 | Beauveria spp |
| 3 | Thevetia neriifolia | Rhizosphere | Soumousso | October-2018 | Beauveria spp |
| 1 | Bombax costatum | Rhizosphere | Soumousso | October-2018 | Beauveria spp |
| 2 | Lagenaria siceraria | Rhizosphere | Soumousso | October-2018 | Beauveria spp |
| 1 | Jatropha curcas | Rhizosphere | Soumousso | October-2018 | Beauveria spp |
| 1 | Hibiscus rosa-sinensis | Rhizosphere | Soumousso | October-2018 | Beauveria spp |
| 2 | Balanites aegyptiaca | Rhizosphere | Soumousso | October-2018 | Beauveria spp |
| 1 | Zea mays | Rhizosphere | Soumousso | October-2018 | Trichoderma spp |
| 1 | Nicotiana tabacum | Rhizosphere | Soumousso | October-2018 | Trichoderma spp |
| 2 | Capsicum frutescens | Rhizosphere | Soumousso | October-2018 | Trichoderma spp |
| 1 | Sorghum bicolor | Rhizosphere | Soumousso | October-2018 | Trichoderma spp |
| 3 | Dioscorea alata | Rhizosphere | Soumousso | October-2018 | Trichoderma spp |
| 2 | Gossypium barbadense | Rhizosphere | Soumousso | October-2018 | Fusarium spp |
| 2 | Vitex donania | Rhizosphere | Soumousso | October-2018 | Aspergilus |
| 2 | Arachis hypogaea | Rhizosphere | Soumousso | October-2018 | Aspergilus |
| 1 | Oryza sativa | Rhizosphere | Soumousso | October-2018 | Aspergilus |
| 2 | Solanum melongena | Rhizosphere | Soumousso | October-2018 | Aspergilus |
| 1 | Ipomoea batatas | Rhizosphere | Soumousso | October-2018 | Aspergilus |
| 1 | Musa paradisiaca | Rhizosphere | Soumousso | October-2018 | Trichoderma spp |
| 1 | Portulaca grandiflora | Rhizosphere | Soumousso | October-2018 | Trichoderma spp |
| 1 | Hibiscus sabdariffa | Rhizosphere | Soumousso | October-2018 | Trichoderma spp |
| 2 | Corchorus olitorius | Rhizosphere | Soumousso | October-2018 | Trichoderma spp |
| 2 | Cymbopogon citratus | Rhizosphere | Soumousso | October-2018 | Trichoderma spp |
| 3 | Acacia ataxacantha | Rhizosphere | Soumousso | October-2018 | ND |
| 1 | PsNDium guajava | Rhizosphere | Soumousso | October-2018 | Trichoderma spp |
| 2 | Phaseolus vulgaris | Rhizosphere | Soumousso | October-2018 | Trichoderma spp |
| 1 | Annona squamosa | Rhizosphere | Soumousso | October-2018 | Trichoderma spp |
| 1 | Calotropopis procera | Rhizosphere | Soumousso | October-2018 | Metarhizium spp |
| 2 | Lannea microcarpa | Rhizosphere | Soumousso | October-2018 | Metarhizium spp |
| 1 | Sesamum indicum | Rhizosphere | Soumousso | October-2018 | Metarhizium spp |
| 1 | Vigna subterranea | Rhizosphere | Soumousso | October-2018 | Metarhizium spp |
| 2 | Vitellaria paradoxa | Rhizosphere | Soumousso | October-2018 | Metarhizium spp |
| 1 | Solanum lycopersicum | Rhizosphere | Soumousso | October-2018 | Metarhizium spp |
| 3 | Thevetia neriifolia | Rhizosphere | Soumousso | October-2018 | Metarhizium spp |
| 2 | Bombax costatum | Rhizosphere | Soumousso | October-2018 | Fusarium spp |
| 2 | Lagenaria siceraria | Rhizosphere | Soumousso | October-2018 | Aspergilus |
| 2 | Jatropha curcas | Rhizosphere | Soumousso | October-2018 | Aspergilus |
| 1 | Hibiscus rosa-sinensis | Rhizosphere | Soumousso | October-2018 | Aspergilus |
| 2 | Balanites aegyptiaca | Rhizosphere | Soumousso | October-2018 | Aspergilus |
| 1 | Zea mays | Rhizosphere | Soumousso | October-2018 | Aspergilus |
| 1 | Nicotiana tabacum | Rhizosphere | Soumousso | October-2018 | Trichoderma spp |
| 2 | Capsicum frutescens | Rhizosphere | Soumousso | October-2018 | Trichoderma spp |
| 1 | Sorghum bicolor | Rhizosphere | Soumousso | October-2018 | Trichoderma spp |
| 2 | Dioscorea alata | Rhizosphere | Soumousso | October-2018 | Trichoderma spp |
| 1 | Gossypium barbadense | Rhizosphere | Soumousso | October-2018 | Trichoderma spp |
| 3 | Vitex donania | Rhizosphere | Soumousso | October-2018 | Trichoderma spp |
| 1 | Arachis hypogaea | Rhizosphere | Soumousso | October-2018 | Trichoderma spp |
| 1 | Oryza sativa | Rhizosphere | Soumousso | October-2018 | Trichoderma spp |
| 2 | Solanum melongena | Rhizosphere | Soumousso | October-2018 | Trichoderma spp |
| 3 | Ipomoea batatas | Rhizosphere | Soumousso | October-2018 | Metarhizium spp |
| 1 | Musa paradisiaca | Rhizosphere | Soumousso | December-2018 | Fusarium spp |
| 2 | Portulaca grandiflora | Rhizosphere | Soumousso | December-2018 | Fusarium spp |
| 1 | Hibiscus sabdariffa | Rhizosphere | Soumousso | December-2018 | Fusarium spp |
| 7 | Corchorus olitorius | Rhizosphere | Soumousso | December-2018 | Fusarium spp |
| 4 | Cymbopogon citratus | Rhizosphere | Soumousso | December-2018 | Fusarium spp |
| 2 | Acacia ataxacantha | Rhizosphere | Soumousso | December-2018 | Fusarium spp |
| 3 | PsNDium guajava | Rhizosphere | Soumousso | December-2018 | Fusarium spp |
| 1 | Phaseolus vulgaris | Rhizosphere | Soumousso | December-2018 | Fusarium spp |
| 1 | Annona squamosa | Rhizosphere | Soumousso | December-2018 | Fusarium spp |
| 2 | Calotropopis procera | Rhizosphere | Soumousso | December-2018 | Fusarium spp |
| 1 | Lannea microcarpa | Rhizosphere | Soumousso | December-2018 | Fusarium spp |
| 1 | Sesamum indicum | Rhizosphere | Soumousso | December-2018 | Aspergilus |
| 2 | Vigna subterranea | Rhizosphere | Soumousso | December-2018 | Aspergilus |
| 1 | Vitellaria paradoxa | Rhizosphere | Soumousso | December-2018 | Aspergilus |
| 1 | Solanum lycopersicum | Rhizosphere | Soumousso | December-2018 | Aspergilus |
| 2 | Thevetia neriifolia | Rhizosphere | Soumousso | December-2018 | Aspergilus |
| 3 | Bombax costatum | Rhizosphere | Soumousso | December-2018 | Trichoderma spp |
| 6 | Lagenaria siceraria | Rhizosphere | Soumousso | December-2018 | Trichoderma spp |
| 4 | Jatropha curcas | Rhizosphere | Soumousso | December-2018 | Trichoderma spp |
| 3 | Hibiscus rosa-sinensis | Rhizosphere | Soumousso | December-2018 | Trichoderma spp |
| 2 | Balanites aegyptiaca | Rhizosphere | Soumousso | December-2018 | Trichoderma spp |
| 1 | Zea mays | Rhizosphere | Soumousso | December-2018 | Trichoderma spp |
| 1 | Nicotiana tabacum | Rhizosphere | Soumousso | December-2018 | Trichoderma spp |
| 2 | Capsicum frutescens | Rhizosphere | Soumousso | December-2018 | Trichoderma spp |
| 1 | Sorghum bicolor | Rhizosphere | Soumousso | December-2018 | ND |
| 1 | Dioscorea alata | Rhizosphere | Soumousso | December-2018 | Fusarium spp |
| 1 | Gossypium barbadense | Rhizosphere | Soumousso | December-2018 | Fusarium spp |
| 3 | Vitex donania | Rhizosphere | Soumousso | December-2018 | Fusarium spp |
| 1 | Arachis hypogaea | Rhizosphere | Soumousso | December-2018 | Fusarium spp |
| 2 | Oryza sativa | Rhizosphere | Soumousso | December-2018 | Fusarium spp |
| 1 | Solanum melongena | Rhizosphere | Soumousso | December-2018 | Fusarium spp |
| 2 | Ipomoea batatas | Rhizosphere | Soumousso | December-2018 | Fusarium spp |
| 1 | Musa paradisiaca | Rhizosphere | Soumousso | December-2018 | Aspergilus |
| 4 | Portulaca grandiflora | Rhizosphere | Soumousso | December-2018 | Aspergilus |
| 1 | Hibiscus sabdariffa | Rhizosphere | Soumousso | December-2018 | Aspergilus |
| 2 | Corchorus olitorius | Rhizosphere | Soumousso | December-2018 | Aspergilus |
| 3 | Cymbopogon citratus | Rhizosphere | Soumousso | December-2018 | Aspergilus |
| 1 | Acacia ataxacantha | Rhizosphere | Soumousso | December-2018 | Trichoderma spp |
| 2 | PsNDium guajava | Rhizosphere | Soumousso | December-2018 | Trichoderma spp |
| 1 | Phaseolus vulgaris | Rhizosphere | Soumousso | December-2018 | Trichoderma spp |
| 7 | Annona squamosa | Rhizosphere | Soumousso | December-2018 | Trichoderma spp |
| 4 | Calotropopis procera | Rhizosphere | Soumousso | December-2018 | Trichoderma spp |
| 2 | Lannea microcarpa | Rhizosphere | Soumousso | December-2018 | Trichoderma spp |
| 3 | Sesamum indicum | Rhizosphere | Soumousso | December-2018 | Trichoderma spp |
| 1 | Vigna subterranea | Rhizosphere | Soumousso | December-2018 | Trichoderma spp |
| 1 | Vitellaria paradoxa | Rhizosphere | Soumousso | December-2018 | Trichoderma spp |
| 2 | Solanum lycopersicum | Rhizosphere | Soumousso | December-2018 | Aspergilus |
| 1 | Thevetia neriifolia | Rhizosphere | Soumousso | December-2018 | Aspergilus |
| 1 | Senna siamea | Rhizosphere | Soumousso | December-2018 | Aspergilus |
| 2 | Lagenaria siceraria | Rhizosphere | Soumousso | December-2018 | Aspergilus |
| 1 | Combretum paniculatum | Rhizosphere | Soumousso | December-2018 | Aspergilus |
| 2 | Saba senegalensis | Rhizosphere | Soumousso | December-2018 | Aspergilus |
| 1 | Vernonia colorata | Rhizosphere | Soumousso | December-2018 | Aspergilus |
| 2 | Musa paradisiaca | Rhizosphere | Soumousso | December-2018 | Aspergilus |
| 1 | Green beans (Phaseolus vulgaris) | Rhizosphere | Soumousso | December-2018 | Beauveria spp |
| 3 | Banana* (Tree) | Rhizosphere | Soumousso | December-2018 | Aspergilus |
| 1 | Zea mays | Rhizosphere | Soumousso | December-2018 | Aspergilus |
| 2 | Abelmoschus esculentus | Rhizosphere | Soumousso | December-2018 | Aspergilus |
| 1 | Oryza sativa ( Asian variety) | Rhizosphere | Soumousso | December-2018 | Aspergilus |
| 1 | Nauclea latifolia | Rhizosphere | Soumousso | December-2018 | Beauveria spp |
| 2 | Green beans (Phaseolus vulgaris) | Rhizosphere | Soumousso | December-2018 | Aspergilus |
| 2 | Zea mays | Rhizosphere | Soumousso | December-2018 | Aspergilus |
| 2 | Sesamum indicum | Rhizosphere | Soumousso | December-2018 | Aspergilus |
| 1 | PsNDium guajava | Rhizosphere | Soumousso | December-2018 | Aspergilus |
| 1 | Vernonia colorata | Rhizosphere | Soumousso | December-2018 | Trichoderma spp |
| 1 | Nauclea latifolia | Rhizosphere | Soumousso | December-2018 | Trichoderma spp |
| 1 | Arachis hypogaea | Rhizosphere | Soumousso | December-2018 | Trichoderma spp |
| 2 | Parkia biglobosa | Rhizosphere | Soumousso | December-2018 | Trichoderma spp |
| 2 | Saba senegalensis | Rhizosphere | Soumousso | December-2018 | Fusarium spp |
| 3 | Phaseolus vulgaris | Rhizosphere | Soumousso | December-2018 | Aspergilus |
| 1 | Oryza sativa ( Asian variety) | Rhizosphere | Soumousso | December-2018 | Aspergilus |
| 2 | Sorghum bicolor | Rhizosphere | Soumousso | December-2018 | Aspergilus |
| 1 | Tamarindus indica | Rhizosphere | Soumousso | December-2018 | Aspergilus |
| 1 | Corchorus olitorius | Rhizosphere | Soumousso | December-2018 | ND |
| 2 | Ipomoea batatas | Rhizosphere | Soumousso | December-2018 | Beauveria spp |
| 1 | Combretum paniculatum | Rhizosphere | Soumousso | December-2018 | Trichoderma spp |
| 1 | Saba senegalensis | Rhizosphere | Soumousso | December-2018 | Trichoderma spp |
| 2 | Vernonia colorata | Rhizosphere | Soumousso | December-2018 | ND |
| 1 | Musa paradisiaca | Rhizosphere | Soumousso | December-2018 | Trichoderma spp |
| 3 | Green beans (Phaseolus vulgaris) | Rhizosphere | Soumousso | December-2018 | Trichoderma spp |
| 2 | Banana* (Tree) | Rhizosphere | Soumousso | December-2018 | Trichoderma spp |
| 2 | Zea mays | Rhizosphere | Soumousso | December-2018 | Trichoderma spp |
| 2 | Jatropha curcas | Rhizosphere | Soumousso | December-2018 | Trichoderma spp |
| 1 | Hibiscus rosa-sinensis | Rhizosphere | Soumousso | December-2018 | ND |
| 2 | Balanites aegyptiaca | Rhizosphere | Soumousso | December-2018 | Trichoderma spp |
| 1 | Zea mays | Rhizosphere | Soumousso | December-2018 | Trichoderma spp |
| 1 | Nicotiana tabacum | Rhizosphere | Soumousso | December-2018 | Trichoderma spp |
| 2 | Senna siamea | Rhizosphere | Soumousso | December-2018 | Trichoderma spp |
| 1 | Sorghum bicolor | Rhizosphere | Soumousso | December-2018 | Trichoderma spp |
| 1 | Dioscorea alata | Rhizosphere | Soumousso | December-2018 | Trichoderma spp |
| 1 | Gossypium barbadense | Rhizosphere | Soumousso | December-2018 | Trichoderma spp |

**
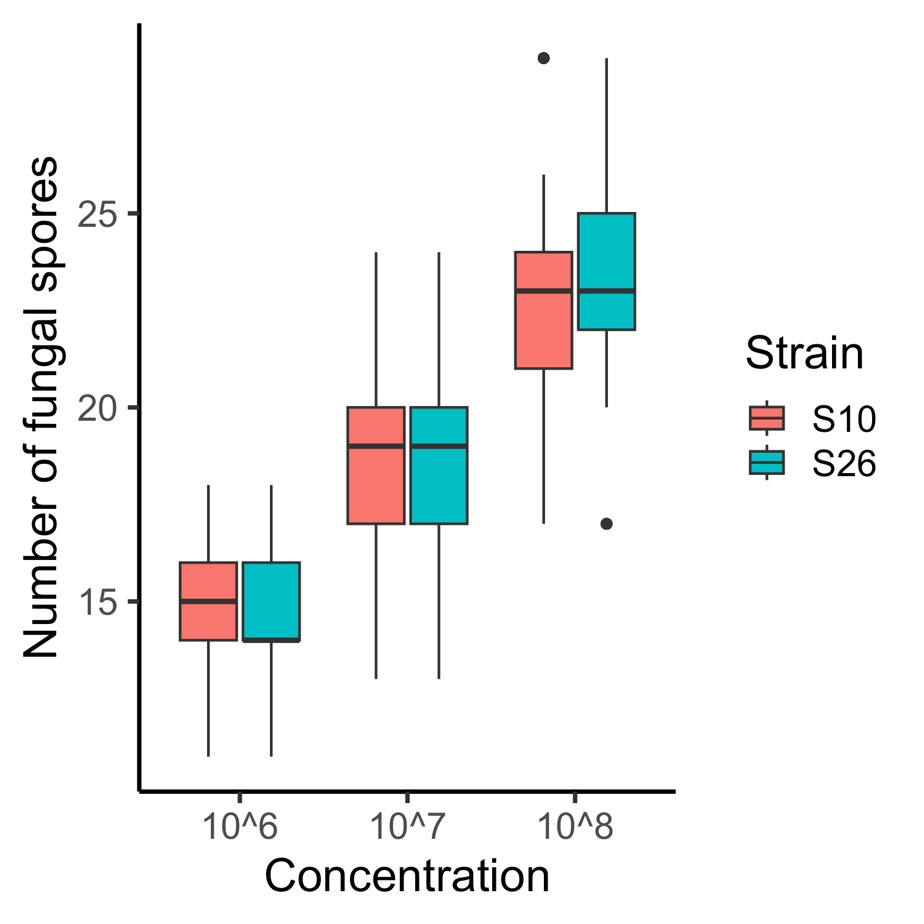
**

**Supplementary Figure 1. Number of spores that indivNDual mosquitoes received for three different concentrations.**

**Supplementary Figure 2: Macroscopic and microscopic features of native strains of Metarhizium pingshaense from Burkina Faso**

**Supplementary Table 2. Statistics for the model on survival after fungal exposure**

| **Type of model** | **Response variable** | **Explanatory variables** | **Chi square** | **P value** | **Hazard ratio** |
| --- | --- | --- | --- | --- | --- |
| Cox proportional hazard | Survival | Fungal treatment | 10.122 | 0.006679 | Control=1  S10=12.5593  S106=6.6318  S26= 8.5465  S43=10.9700 |
|  |  | Concentration | 460.53 | < 2.2^-16^ | 10^6^=1  10^7^=0.8299  10^8^=1.0141 |

**Supplementary Table 3. Statistics for the model on association between fungal exposure time and strain.** * denotes an interaction between two explanatory variables.

| **Type of model** | **Response variable** | **Explanatory variables** | **Deviance** | **P value** | **Coefficients** (logit) |
| --- | --- | --- | --- | --- | --- |
| General linear model (binomial distribution) | Fungal growth | Fungal strain * Time | -9.7789 | 0.02054 | S10-10^6^-1h = -23.86  S26-10^6^-1h = -18.84  S43-10^6^-1h = -18.84  S106-10^6^-1h = -18.84  24h = 22.25  S26*24h = 18.05  S43*24h = 19.56  S106*24h = 18.84 |
|  |  | Concentration | -178.93 | < 2.2^-16^ | 10^8^ = 22.25 |

**Supplementary Table 4. Statistics for the model on survival after exposure to soil isolated fungi or fungal infected mosquitoes**

| **Type of model** | **Response variable** | **Explanatory variables** | **Chi square** | **P value** | **Hazard ratio** |
| --- | --- | --- | --- | --- | --- |
| Cox proportional hazard | Survival | Fungal treatment | 275.76 | 2.2^-16^ | S26 = 1  S10 = 12.0048  S106 = 5.9840  S43= 11.5458 |
|  |  | Origin | 41.342 | 1.105^-10^ | Mosquito = 1  Soil = 0.05679 |
